# Cellular Aging and Senescence in Cancer: A Holistic Review of Cellular Fate Determinants

**DOI:** 10.14336/AD.2024.0421

**Published:** 2025-05-21

**Authors:** Muhammad Tufail, Yu-Qi Huang, Jia-Ju Hu, Jie Liang, Cai-Yun He, Wen-Dong Wan, Can-Hua Jiang, Hong Wu, Ning Li

**Affiliations:** ^1^Department of Oral and Maxillofacial Surgery, Center of Stomatology, Xiangya Hospital, Central South University, Changsha, China.; ^2^Institute of Oral Precancerous Lesions, Central South University, Changsha, China.; ^3^Research Center of Oral and Maxillofacial Tumor, Xiangya Hospital, Central South University, Changsha, China.; ^4^National Clinical Research Center for Geriatric Disorders, Xiangya Hospital, Central South University, Changsha, China.; ^5^State Key Laboratory of Powder Metallurgy, Central South University, Changsha, China

**Keywords:** Cellular Aging, Senescence, Cancer Progression, Senescence Escape, Therapeutic Strategies

## Abstract

This comprehensive review navigates the complex relationship between cellular aging, senescence, and cancer, unraveling the determinants of cellular fate. Beginning with an overview of cellular aging's significance in cancer, the review explores processes, changes, and molecular pathways influencing senescence. The review explores senescence as a dual mechanism in cancer, acting as a suppressor and contributor, focusing on its impact on therapy response. This review highlights opportunities for cancer therapies that target cellular senescence. The review further examines the senescence-associated secretory phenotype and strategies to modulate cellular aging to influence tumor behavior. Additionally, the review highlights the mechanisms of senescence escape in aging and cancer cells, emphasizing their impact on cancer prognosis and resistance to therapy. The article addresses current advances, unexplored aspects, and future perspectives in understanding cellular aging and senescence in cancer.

## 1. Introduction

Cellular aging is the natural process of cells deteriorating over time, leading to reduced function and increased susceptibility to disease. Cellular aging has a profound role in shaping the fate of cells, transcending mere temporal boundaries to orchestrate a complex symphony of molecular events. From the intricate dynamics of telomeres to the precise choreography of genomic maintenance, the aging process represents a delicate interplay of molecular intricacies. As cells journey through time, they accumulate a spectrum of age-related changes, rendering them susceptible to protective and detrimental influences [1, 2]. Understanding the paradigm of cellular aging is pivotal in unraveling the subsequent chapters on senescence and cancer, as it lays the foundation for balancing cellular preservation and transformation.

Exploring cellular aging in cancer unveils the intricate relationship between these phenomena [3]. Cancer, arising from cellular dysregulation, presents a significant challenge within the framework of aging cells. This exploration extends beyond academic realms; it holds promise in revealing therapeutic strategies targeting age-associated vulnerabilities in cancer [4, 5]. This thorough review provides a foundation for reshaping perspectives on aging-related diseases, particularly cancer, by offering insights into potential therapeutic interventions.

At the nexus of cellular aging lies senescence, a stable cell cycle arrest state with multifaceted implications for cellular homeostasis. This section examines the dynamic interplay between cellular aging and senescence, examining how the aging process shapes the cellular landscape, fostering conditions that may either inhibit or propel senescence. The tapestry extends to the profound connection between senescence and cancer, where cells are entangled in a delicate balance of protection and peril [6]. Understanding this link is paramount, as it reveals the dual role senescence plays both as a defender against tumorigenesis and, paradoxically, as a contributor to cancer progression.

As we navigate this holistic review, the significance of investigating cellular aging within the context of cancer comes into sharp focus. Cancer, a manifestation of cellular dysregulation, represents a formidable challenge within the framework of aging cells. Unraveling the intricate relationship between cellular aging and cancer susceptibility, the role of senescence as a sentinel against tumorigenesis, and the deviation from this protective mechanism becomes crucial [7]. This exploration is not merely an academic pursuit; it promises to uncover therapeutic strategies targeting age-associated cancer vulnerabilities [8]. Understanding the determinants of cellular fate in this holistic manner lays the groundwork for redefining our perspectives on aging-related diseases, particularly cancer. We will traverse the realms of senescence, apoptosis, and cancer therapies, intricately examining their roles as cellular fate determinants within the broader context of aging. This holistic review aims to provide a nuanced understanding of cellular destiny's molecular intricacies, offering insights that may reshape our paradigms in both basic cellular biology and the clinical realm of age-related cancer.

## 2. Cellular Aging: A Prelude to Senescence and Cancer

### 2.1. Processes Involved in Cellular Aging

Cellular senescence is triggered by various cellular challenges, including DNA damage [9, 10], telomere shortening [11, 12], and oncogene activation [13]. These factors initiate a cascade of events that safeguard against cancer during early life and actively contribute to the aging process. For instance, the cellular senescence program is activated when cells undergo DNA damage due to external factors like radiation or chemicals. This response halts the replication of damaged cells, preventing the potential propagation of mutations and, consequently, acting as a critical defense against cancer initiation [14, 15]. Telomere shortening, a consequence of replication-associated telomere shortening, can induce replicative senescence by activating DNA damage response signaling pathways. Telomere shortening triggers replicative senescence by inducing DNA damage response signaling pathways. This process involves the activation of two major tumor-suppressor pathways, the p53/p21^Waf1/Cip1^ and p16^Ink4a^/Rb pathways, leading to an irreversible cell cycle arrest, usually in the G1 phase [16]. On the other hand, oncogene activation drives cellular senescence by engaging robust tumor suppressive processes that recognize and counteract inappropriate proliferative signals. This includes directing cells with abnormal growth signals toward cell death or an irreversible cell cycle arrest known as cellular senescence [17]. DNA damage and telomere shortening emerge as hallmark features of cellular aging, intricately linked to the initiation of cellular senescence. Consider the scenario of telomere shortening with each cell division; the telomeres, protective caps at the ends of chromosomes, progressively shorten. This natural biological clock eventually triggers cellular senescence, limiting the replicative potential of cells and contributing to the overall aging landscape within tissues [18, 19].

The tumor-suppressive role of senescence is noteworthy in its ability to impede cancer progression. Senescence induces a robust cell cycle arrest, preventing the uncontrolled division of cells and acting as a sentinel against the progression from pre-malignant to malignant states. This mechanism ensures that potentially harmful cells are halted in their tracks, safeguarding against tumorigenesis [20]. However, the dual role of senescence in cancer adds a layer of complexity. While senescence is a defense mechanism against tumorigenesis, the persistent presence of senescent cells may paradoxically contribute to cancer progression [21]. The senescence-associated secretory phenotype (SASP) is a characteristic feature of senescent cells, where these cells secrete various factors such as cytokines, growth factors, and proteases. These secreted factors can influence the surrounding microenvironment, promoting inflammation and tissue remodeling, and potentially contributing to age-related diseases. The secretory profile of senescent cells, known as the SASP, can create a pro-inflammatory microenvironment, fostering conditions conducive to tumorigenesis. This delicate balance between protective and potentially harmful effects underscores the nuanced nature of senescence in cancer development [22, 23].

The interplay between aging and cancer becomes more apparent through inflammatory connections. Through the SASP, Senescent cells actively contribute to tissue degeneration and may promote tumorigenesis by inducing inflammation. Chronic inflammation induced by senescent cells in aged tissues exemplifies how these processes are intertwined, shaping the microenvironment and influencing aging-related changes and cancer development [6, 24]. For example, Systemic inflammation and aging can lead to a condition called CHIP (clonal hematopoiesis of indeterminate potential), which has implications for cancer development. CHIP refers to a condition characterized by genetically distinct populations of blood cells derived from a single hematopoietic stem cell. These cells harbor somatic mutations commonly associated with hematologic malignancies but do not meet diagnostic criteria for blood cancer. CHIP increases with age and is associated with an elevated risk of developing blood cancers, cardiovascular disease, and overall mortality.

### 2.2. The Impact of Cellular Aging on Senescence

As cells undergo aging, the intricate relationship between cellular aging and the tumor-suppressive role of senescence becomes evident [25]. Senescence, functioning as a robust defense mechanism, is crucial in halting cancer cell proliferation and impeding the progression from pre-malignant to malignant states. This dynamic interplay sheds light on how cellular aging actively contributes to the orchestration of senescence, forming a critical component in preventing tumorigenesis [25]. Activated oncogenes, which promote uncontrolled cell growth, trigger senescence as a countermeasure. This process is a formidable barrier against cancer development by preventing the growth of potentially neoplastic cells [26]. The delicate balance between cellular aging and the activation of oncogenes highlights the sophisticated interplay between these processes and their collective impact on cellular health [27, 28].

The innate immune system targets senescent cells for elimination, emphasizing its crucial role in regulating the presence of senescent cells through immune surveillance. This immune response adds complexity to understanding how cellular aging influences senescence and, consequently, shapes the landscape of cancer development [29, 30]. For example, the interactions between senescent cells and the immune system are crucial in regulating cellular aging and cancer susceptibility. Immune surveillance mechanisms target senescent cells for elimination, highlighting the importance of immune responses in controlling the presence of senescent cells. Factors like the clearance of senescent cells by immune cells, immune surveillance promoting senescence induction and clearance, the impact of the SASP on immune responses, and emerging senolytic immunotherapy (immunotherapy that targets and eliminates senescent cells using the immune system to treat age-related diseases) approaches underscore the complex relationship between cellular aging, immune regulation, and cancer development [31].

**Table. 1:** Senescence in Cancer: Pros and Cons.

*Aspect of Senescence*	*Positive Effect in Cancer*	*Negative Effect in Cancer*
***Growth Arrest***	Senescence induces a permanent growth arrest in cancer cells, preventing uncontrolled proliferation and metastasis. This halts tumor growth and can lead to tumor dormancy.	Senescent cells can promote tumor progression through the secretion of various factors, such as pro-inflammatory cytokines and growth factors that stimulate neighboring cancer cells, fostering a pro-tumorigenic microenvironment and aiding in tumor survival and spread.
***Immune Surveillance***	Senescent cancer cells are recognized and cleared by the immune system, reducing the tumor burden. This immune surveillance helps in controlling cancer growth and preventing metastasis.	Some senescent cells may evade immune detection through mechanisms like upregulation of immune checkpoint molecules, leading to immune tolerance and allowing tumor cells to escape immune destruction, contributing to cancer progression.
***Tumor Suppression Pathways***	Senescence activates tumor-suppressive pathways, such as p53 and pRB, inhibiting cancer initiation and progression. This serves as a natural barrier against tumorigenesis.	Senescent cells can secrete factors like matrix metalloproteinases, cytokines, and growth factors that create a pro-tumorigenic microenvironment, promoting tumor growth, angiogenesis, and invasion, ultimately aiding in tumor progression.
***Therapy Resistance***	Certain cancer treatments, like chemotherapy and radiation therapy, can trigger senescence in cancer cells, halting tumor growth and contributing to treatment efficacy.	Senescent cells may confer resistance to therapy by promoting the survival of cancer cells under treatment-induced stress. They can secrete factors that enhance cell survival pathways, leading to therapy resistance and potentially allowing residual cancer cells to repopulate the tumor.

In therapeutic implications, the discourse focuses on strategies targeting senescence in cancer therapy. Notably, therapy-induced senescence emerges as a promising approach. This strategy aims to prevent tumor growth by inducing senescence in cancer cells while minimizing potential side effects. Understanding how cellular aging influences therapeutic responses provides valuable insights into novel avenues for cancer treatment, ushering in a more nuanced and effective approach to tackling this complex disease.

## 3. Senescence: A Double-Edged Sword in Cancer

### 3.1. Definition and Characteristics of Senescence

Originally characterized as a physiological suppressor mechanism against tumor cells due to its ability to halt cell proliferation, senescence plays a pivotal role in the prevention and progression of cancer (Table. 1). Its initial role as a tumor suppressor is grounded in the ability to enforce a halt in cell division, preventing the unchecked growth of potential malignancies [32]. However, recent insights have brought forth complexities. Senescent cells may contribute to oncogenesis through mechanisms like the SASP [33]. For example, the SASP may create a microenvironment that promotes tumor recurrence or progression, demonstrating the dualistic nature of senescence in cancer.

Senescence establishes intricate connections with various anticancer therapies, such as chemotherapy, radiotherapy, and targeted therapies [34]. Its influence is dual-fold. On one hand, senescence can augment the effectiveness of treatments by directly inhibiting cancer cell proliferation or by eliciting an immune response through bioactive molecules released by senescent cells. On the other hand, there's a potential downside, where senescence may compromise patient resilience to therapies, potentially setting the stage for disease recurrence post-treatment [34, 35]. For instance, chemotherapy induces senescence in both tumors and normal cells. Low doses of chemotherapy can trigger a senescent state in human cancer cells, while higher doses may induce apoptosis instead [36]. Furthermore, targeted therapies can also induce senescence in cancer cells. Activating oncogenes like HRAS^V12^ can trigger growth arrest, known as oncogene-induced senescence [36].

Senescent cells exhibit an increased prevalence in the normal tissues of aged individuals and specific tissue types like skin and adipose tissue. The presence of senescent cells in cancer patients holds profound prognostic implications, influencing outcomes that range from improved to impaired [37, 38]. The varying effects of senescence on tumorigenesis and response to therapy underscore its intricate role in shaping the trajectory of cancer outcomes [39]. For instance, the prevalence of senescent cells in the tumor microenvironment may significantly impact the response to treatment, leading to diverse outcomes based on the complex dynamics of senescence in influencing cancer progression and therapeutic responses.

Viewing cellular senescence as a dynamic, evolving condition in the context of cancer is paramount [40]. Senescence exhibits both antitumorigenic and protumorigenic features that can shift over time. The interactions between senescent cells and the tumor microenvironment add a layer of complexity to its role in cancer progression and treatment [41]. As an illustration, senescent cells may initially contribute to the suppression of tumors but may later foster conditions conducive to tumor progression. Recognizing this dynamic nature is crucial for developing nuanced and effective strategies that leverage the positive aspects of senescence while mitigating its potentially detrimental effects on cancer outcomes [42].

### 3.2. Senescence as a Tumor Suppressive Mechanism

**Figure1.**
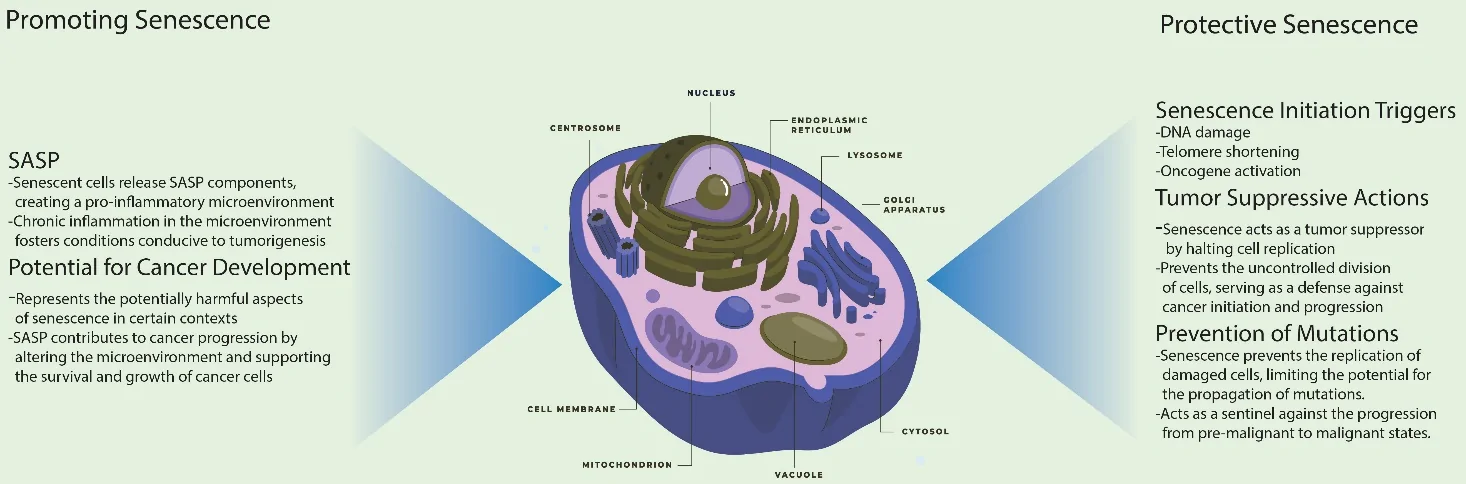
**This figure illuminates the intricate role of senescence in cancer development, portraying a dual scenario: Protective Senescence and Promoting Senescence**. The initiation triggers of senescence, encompassing DNA damage, telomere shortening, and oncogene activation, activate a protective response, preventing the propagation of mutations. Senescence acts as a robust tumor suppressor by halting cell replication, defending against uncontrolled cell division, and acting as a sentinel against cancer initiation and progression. Additionally, senescence prevents the replication of damaged cells, limiting the potential for mutations and impeding the progression from pre-malignant to malignant states. On the other hand, the potentially harmful aspects of senescence are depicted. Senescent cells release the SASP, creating a pro-inflammatory microenvironment. Chronic inflammation in this microenvironment fosters conditions conducive to tumorigenesis, representing the dark side of senescence. This side illustrates the potential for cancer development as SASP contributes to cancer progression by altering the microenvironment and providing support for the survival and growth of cancer cells.

In Cellular Senescence and Cancer Suppression, senescence activation responds to various triggers such as DNA damage, telomere shortening, and oncogene activation. Its significance lies in acting as a robust barrier against cancer by initiating growth arrest in damaged cells. This halts their uncontrolled proliferation, thwarting the potential transformation into malignant cells (Fig. 1) [43]. For instance, in response to DNA damage caused by external factors, the induction of senescence prevents the replication of genetically compromised cells, thereby impeding cancer initiation.

The Paradoxical Role of Senescence introduces a nuanced perspective. While senescence is traditionally seen as a safeguard by inhibiting the proliferation of potentially harmful cells, a paradoxical aspect surface. Senescent cells may exhibit a SASP that, counterintuitively, promotes tumor growth, invasion, metastasis, and vascularization. This paradox underscores the complexity of senescence in the intricate landscape of cancer biology. An illustrative example is the SASP-mediated secretion of inflammatory molecules, creating a microenvironment conducive to tumor progression [2].

Moving to Therapeutic Implications, senescence induced by chemotherapy, radiotherapy, and targeted therapies stands out as a beneficial facet of cancer treatment. It inhibits cell proliferation and stimulates an immune response against cancer cells [44]. However, accumulating senescent cells and SASP components may pose risks over time, necessitating a nuanced approach. For instance, chemotherapy-induced senescence can enhance immediate treatment success but may contribute to long-term complications such as therapy resistance.

Regarding Clinical Relevance, senescent cells become prevalent in aged tissues and wield significance in cancer prognosis and treatment outcomes [1]. Identifying senescent cells through specific biomarkers like senescence-associated β-galactosidase, p21, and p16INK4A enables researchers to explore the prognostic implications of senescence in cancer patients [45]. This identification aids in understanding how the presence of senescent cells may influence the course of cancer and response to treatment.

The dynamic nature of senescence in cancer therapy calls for further research. Understanding the circumstances under which senescent cells exert beneficial or detrimental effects on treatment outcomes is paramount. Strategies involving the selective removal of senescent cells or the modulation of SASP effects present promising avenues for enhancing the efficacy of cancer therapies while minimizing potential risks associated with senescence-induced tumorigenesis. For example, exploring therapeutic interventions that selectively target senescent cells without compromising the overall benefits of senescence in cancer treatment holds promise for future advancements in oncology.

### 3.3. Senescence as a Contributor to Cancer Development

Initiated by diverse stressors such as DNA damage, telomere shortening, and oncogene activation, cellular senescence acts as a stress response mechanism [46]. Its protective role is evident as it halts the proliferation of damaged cells, preventing uncontrolled growth (Fig. 1) [47]. However, the paradox unfolds through the SASP, where senescent cells release factors promoting tumor growth, invasion, metastasis, and vascularization [21]. For instance, in response to telomere shortening, senescence prevents cells from dividing further, averting potential tumorigenesis [21].

The role of senescence in cancer is both intricate and controversial. Traditionally recognized as a tumor suppressor mechanism due to its ability to curb uncontrolled cell proliferation, recent evidence challenges this notion [22]. Senescent cells may paradoxically foster oncogenesis, mainly through SASP-mediated effects [48]. The accumulation of senescent cells and SASP components over time raises susceptibility to tumorigenesis, portraying the dual nature of senescence in cancer biology [21]. An example is seen in oncogene activation, where senescence may initially act as a defense but later contribute to a microenvironment conducive to tumor progression [46].

Senescence induced by chemotherapy, radiotherapy, or targeted therapies presents a double-edged sword in cancer treatment outcomes [22]. While it can inhibit cell proliferation and boost immune responses against cancer cells, the persistence of senescent cells and SASP components may lead to unintended consequences such as tumor recurrence or progression. Strategies that selectively target or remove senescent cells promise to optimise cancer therapy outcomes. For instance, in chemotherapy, inducing senescence may hinder immediate cancer growth but necessitates a nuanced approach to mitigate long-term risks [22].

Further research becomes imperative to delineate the precise conditions under which senescent cells exert beneficial or detrimental effects on cancer development and treatment outcomes. Strategies focusing on modulating the effects of SASP or selectively targeting senescent cells represent promising avenues for enhancing the efficacy of cancer therapies while minimizing potential risks associated with senescence-induced tumorigenesis. For instance, exploring interventions that selectively target or modulate SASP components may provide tailored approaches in cancer therapeutics.

**Figure 2.**
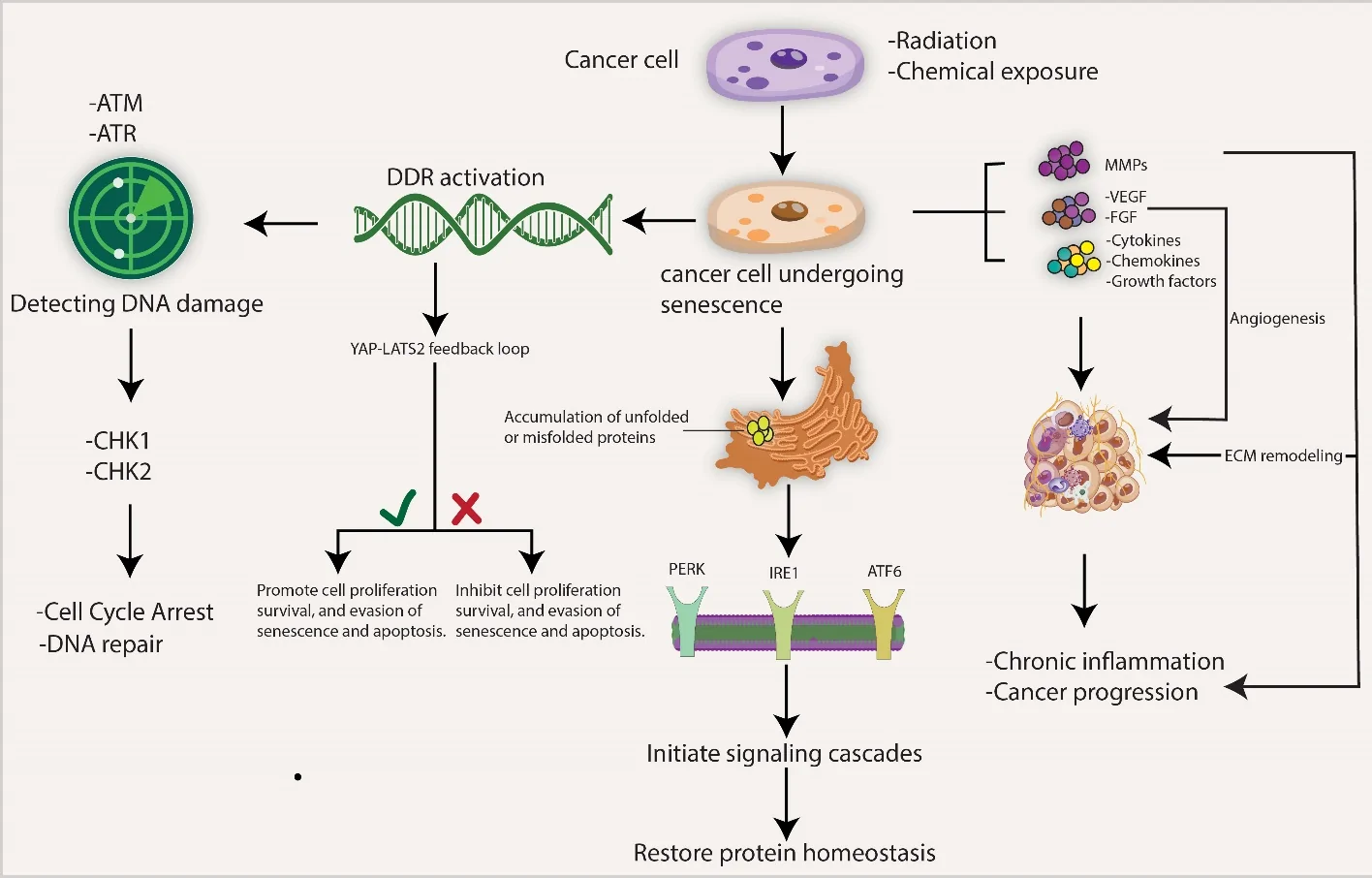
**This illustrative figure portrays the dynamic regulation of cellular senescence in a cancer cell through the pathways**. The cancer cell undergoing senescence is highlighted, featuring a pronounced nucleus and chromatin. Sources of DNA damage, including radiation and chemicals, surround the cell, initiating DDR. ATM and ATR, depicted as vigilant sentinel proteins, detect damage, transmitting signals downstream to activated gears or switches, CHK1 and CHK2. The bottom section illustrates DDR-induced cell cycle arrest at checkpoints like G1 and G2, along with active DNA repair. This figure also illustrates the intricate interplay between the UPR pathway and senescence regulation in cancer cells. The cancer cell undergoing senescence is depicted. The accumulation of unfolded or misfolded proteins within the endoplasmic reticulum (ER) activates the UPR pathway. Three main sensors of the UPR—PERK, IRE1, and ATF6—detect unfolded proteins and initiate signaling cascades to restore protein homeostasis. In the middle section, the UPR pathway regulates protein synthesis, folding, and degradation to alleviate ER stress and prevent the accumulation of misfolded proteins. UPR activation in the context of senescence aims to manage protein quality control, ensuring proper cellular function and preventing proteotoxic stress-induced senescence. Senescent cells release SASP factors, including inflammatory cytokines, chemokines, and growth factors, which recruit immune cells and induce senescence in neighboring cells, contributing to chronic inflammation and cancer progression. Moreover, senescent cells secrete MMPs as part of the SASP, leading to ECM remodeling characterized by collagen degradation and alterations in tissue structure. Furthermore, senescent cells modulate angiogenesis within the tumor microenvironment by secreting factors such as VEGF and FGF, promoting blood vessel formation and tumor growth. Also, Feedback loops between senescence and cancer-promoting pathways, exemplified by the YAP-LATS2 feedback loop, further influence cancer development.

### 3.4 Molecular Signaling Pathways Regulating Senescence in Cancer

In the landscape of cancer, the molecular signaling pathways orchestrating senescence represent a complex interplay that profoundly influences tumor development, progression, and responses to therapeutic interventions (Fig. 2). Senescence induction in cancer is intricately regulated by a myriad of triggers, including DNA damage, telomere shortening, oncogene activation, and other cellular stressors [49]. These triggers activate several signaling pathways, such as the DNA damage response (DDR), cell cycle regulation machinery, apoptosis regulation, cellular energy metabolism, and the unfolded protein response (UPR) [46]. The DDR pathway is a critical cellular mechanism triggered by DNA damage from various sources [50]. This pathway involves detecting DNA damage by sensor proteins like ATM and ATR, activating downstream effectors such as CHK1 and CHK2 to halt the cell cycle. Subsequently, DNA repair processes are initiated to maintain genomic stability [51, 52]. In the context of senescence, DDR activation plays a crucial role in inducing cell cycle arrest, preventing the propagation of damaged cells, and promoting senescence as a tumor-suppressive mechanism [50, 51]. This process involves the establishment of cellular senescence, where cells remain alive but permanently unable to further proliferate, contributing to both aging and cancer research [53]. The DDR signaling pathway is intricately linked to cellular senescence through mechanisms involving oncogene activation, irreparable DNA damage, and the activation of various proteins like ATM, ATR, p53, and p21 to regulate cell cycle progression and maintain genomic integrity [54]. The UPR pathway is a cellular signaling pathway that responds to the accumulation of unfolded or misfolded proteins in the endoplasmic reticulum (ER), a cellular organelle involved in protein synthesis and folding. The UPR pathway aims to restore ER homeostasis by increasing the capacity of the ER to fold and degrade proteins or by reducing the load of protein synthesis. While the UPR pathway is activated in response to the accumulation of unfolded or misfolded proteins in the endoplasmic reticulum. This triggers the activation of sensors like PERK, IRE1, and ATF6, which initiate pathways regulating protein synthesis, folding, and degradation to restore protein homeostasis [55]. In senescence, UPR activation aims to manage protein quality control, ensuring proper cellular function and preventing proteotoxic stress-induced senescence [56]. The UPR pathway represents a critical signaling platform associated with major senescence hallmarks, highlighting its role in maintaining cellular integrity and function during aging and stress-induced conditions [57, 58]. Collectively, these pathways culminate in the initiation of senescence, a phenomenon that can either impede or, paradoxically, contribute to cancer progression.

Regulatory feedback loops are mechanisms within biological systems where the output of a process influences the input or activity of the same process, creating a self-regulating cycle. These loops play a fundamental role in maintaining stability, homeostasis, and adaptability in various biological processes. Regulatory feedback loops form a crucial aspect of senescence's influence on cancer. These loops within key cellular pathways interplay with immune modulation, inflammation, extracellular matrix maintenance, and angiogenesis [59, 60]. In the context of immune modulation, senescent cells can impact immune surveillance and clearance mechanisms, leading to their accumulation within aging tissues. This process is associated with the pro-inflammatory phenotype, the SASP, which can have tumor-suppressive functions and promote immune activation [61]. The mechanism behind this process involves the secretion of SASP factors by senescent cells. These factors can induce senescence in surrounding cells and promote a pro-inflammatory environment [62]. Moreover, recent studies have highlighted the role of innate immune responses, particularly the cGAS-STING pathway, in triggering SASP induction [63]. The cGAS-STING pathway is a crucial signaling pathway in the innate immune response to cytoplasmic DNA. It is critical in detecting and responding to cellular stress, infection, and DNA damage. This pathway is crucial for regulating cellular senescence and the associated pro-inflammatory phenotype. Understanding and regulating SASP may offer insights into managing age-associated diseases and cancer progression [31]. Meanwhile, the senescent cells release inflammatory factors through the SASP, contributing to chronic inflammation and influencing cancer development. Strategies like senolysis aim to eliminate senescent cells to control SASP effects [62, 63]. Moreover, Senescent cells impact the extracellular matrix by secreting factors like matrix metalloproteinases, which play a role in remodeling matrix components such as collagen. The mechanism involves senescent cells releasing these enzymes as part of the SASP. Matrix metalloproteinases are known for their ability to degrade various components of the extracellular matrix, including collagen, leading to alterations in tissue structure and function. This process contributes to the age-related changes observed in tissues and can have implications for conditions like cancer progression [63]. Also, Senescent cells can influence angiogenesis, a process crucial for tumor growth, by secreting factors that modulate blood vessel formation and remodeling in the tumor microenvironment. The mechanism behind this involves the SASP, where senescent cells secrete a variety of proteins, cytokines, and growth factors that can alter the local tissue environment. These secreted factors from senescent cells induce senescence in surrounding cells and play a role in promoting chronic inflammation and cancer progression [5, 63]. Another example is the interruption of the YAP-LATS2 feedback loop causes ovarian cells to transition from YAP-induced senescence to malignant transformation [64]. The YAP-LATS2 feedback loop is a regulatory mechanism involved in the Hippo signaling pathway, which plays a critical role in controlling cell growth, proliferation, and organ size regulation. While the precise nature of these interactions remains incompletely understood, their significance in shaping the impact of senescence on tissue structure and function holds substantial implications for cancer development and treatment outcomes.

The SASP emerges as a pivotal mediator of the effects of senescent cells on cancer progression. SASP components can induce or enhance growth arrest in autocrine and paracrine manners, inhibiting cancer progression [65]. Autocrine signaling occurs when a cell releases signaling molecules that bind to receptors on its surface, essentially signaling itself. This mechanism is often involved in processes such as cellular growth and repair. Paracrine signaling, conversely, involves cells releasing signaling molecules into the extracellular fluid, affecting nearby cells rather than the cell that secreted them. This signalling mode is crucial for coordinating activities among neighboring cells, playing roles in immune responses and neurotransmission processes. Simultaneously, however, SASP factors may also promote cell proliferation, angiogenesis, aging, tumorigenesis, and metastasis, underscoring the dual nature of senescence in cancer biology [66]. Examples include interleukins, chemokines, and matrix metalloproteinases within the SASP, illustrating its diverse impact on the tumor microenvironment.

Senescence induced by cancer therapies, such as radiotherapy and chemotherapy, poses a unique challenge. While therapy-induced senescence may initially exhibit a cytostatic clinical response, there is a risk of these senescent cells reactivating and contributing to tumor recurrence or progression [49, 67]. The molecular underpinnings of therapy-induced senescence are critical for optimizing treatment strategies and enhancing long-term tumor control. For instance, understanding the intricate balance between senescence and cell death pathways could inform therapeutic regimens to prevent tumor relapse [34, 68].

## 4. Senescence and Cancer Therapies

### 4.1. The Role of Senescence in Response to Cancer Treatments

In the intricate tapestry of cancer therapies, the interplay between senescence and treatment outcomes emerges as a complex dynamic that significantly influences the effectiveness of interventions, tumor response patterns, and the resilience of patients [66]. Cancer treatments such as chemotherapy, radiotherapy, and endocrine therapies exhibit the capability to induce cellular senescence, imposing a state of stable growth arrest in cancer cells. This induction of senescence represents a crucial mechanism through which these treatments exert their cytostatic effects, effectively inhibiting the proliferation of cancer cells and, in some cases, enhancing immune responses against malignant cells [66]. For example, radiotherapy-induced senescence has been observed in various cancer types, including breast cancer, where irradiation triggers the DNA damage response, leading to the activation of senescence programs.

While therapy-induced senescence contributes to the initial success of treatments by halting cancer cell growth, a dual nature of senescence emerges in the context of cancer therapy. Senescent cells, in addition to their growth-arrested state, may exhibit pro-aging impacts by releasing bioactive molecules via the SASP [36]. This complex process stimulates an immune response against cancer cells and potentially undermines patient resilience to cancer therapies. This phenomenon increases the risk of disease recurrence post-treatment, emphasizing the need for a nuanced understanding of senescence in the context of therapeutic interventions [69].

**Figure 3.**
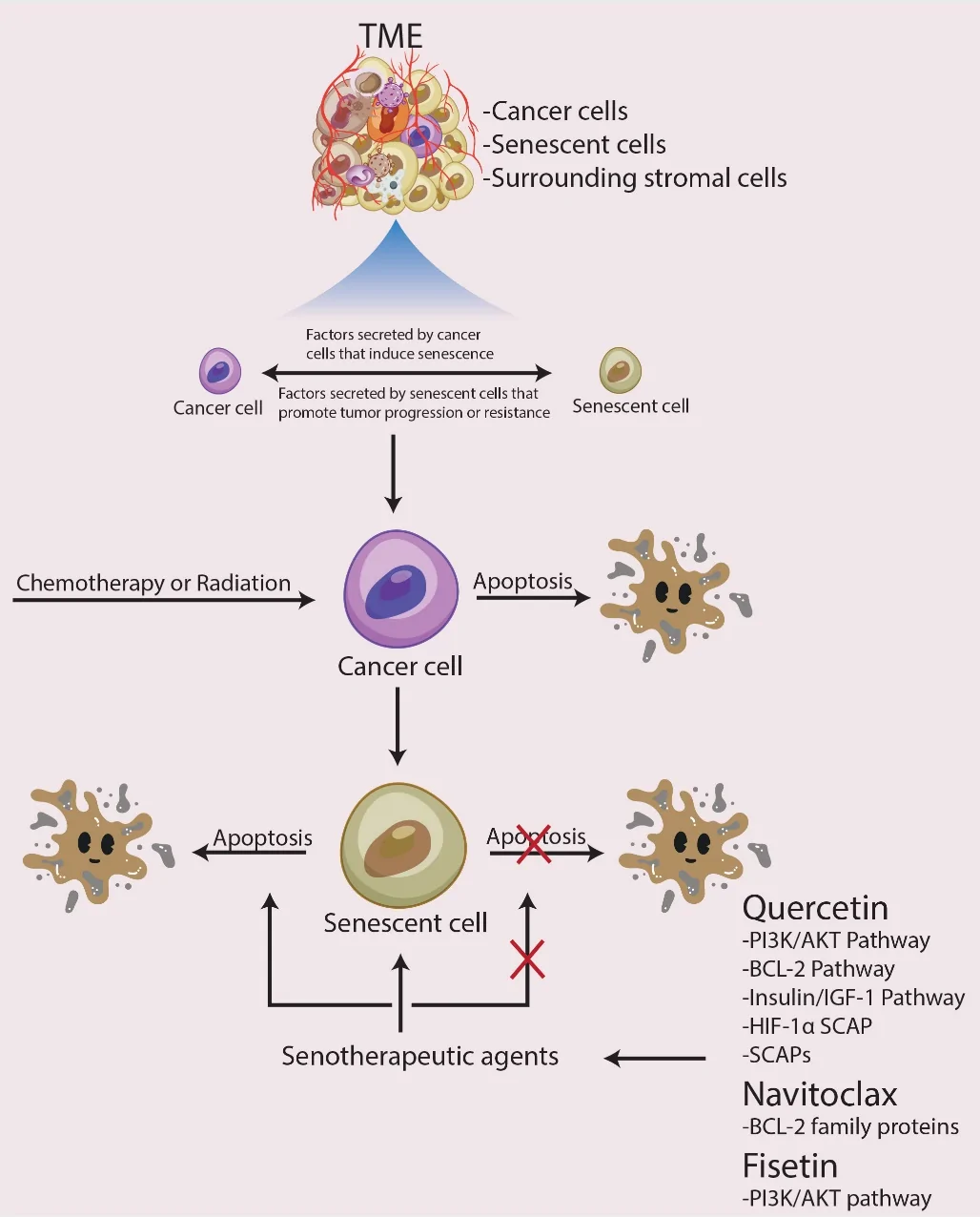
**In this figure, the tumor microenvironment is depicted with cancer cells, senescent cells, and stromal cells**. Bidirectional arrows illustrate the interplay between senescence and cancer cells, indicating factors secreted by both cell types that influence tumor progression and treatment response. Senotherapeutic agents, including quercetin, navitoclax, and fisetin are depicted and accompanying descriptions of their mechanisms of action. Arrows connecting these compounds to senescent cells illustrate their ability to target and eliminate senescent cells, thereby enhancing treatment efficacy.

The advent of senotherapies, encompassing senolytic drugs that selectively target and eliminate senescent cells or senostatic drugs that inhibit their function, introduces a novel approach to enhance the efficacy of cancer therapies (Fig. 3). For instance, compounds like quercetin, navitoclax, and fisetin are actively under investigation for their potential roles in improving treatment outcomes by mitigating the pro-aging impacts of senescent cells [70, 71]. Quercetin, a flavonoid compound, has shown promise in improving treatment outcomes by targeting senescent cells. It acts as a senolytic agent, promoting the selective elimination of senescent cells. The mechanism of quercetin involves disrupting multiple pathways associated with senescence, leading to the clearance of these dysfunctional cells. Quercetin has potent senolytic effects when combined with dasatinib, enhancing health and lifespan by eliminating age-related senescent cells [72, 73]. Navitoclax, another compound under investigation for its role in mitigating the pro-aging impacts of senescent cells, induces thrombocytopenia. This drug targets BCL-2 family proteins, promoting apoptosis in senescent cells. Navitoclax reduces the burden of dysfunctional cells by inducing programmed cell death, which could enhance treatment outcomes in age-related conditions [74]. Fisetin, a flavonoid similar to quercetin, exhibits cell-type-specific senolytic properties by altering multiple pathways associated with senescence. It has diverse mechanisms of action on senescent cells and has been shown to promote apoptosis in these cells. Fisetin's mechanism may involve blocking the PI3K/AKT pathway, eliminating senescent cells, and potentially extending health and lifespan. Studies have highlighted fisetin's ability to induce apoptosis in lung cancer cells through various mechanisms, including reducing anti-apoptotic proteins like BCL-2 [75, 76]. These drugs showcase the evolving landscape of cancer therapeutics, aiming to refine treatment strategies and mitigate the challenges of senescence-induced effects [77, 78].

Further research becomes imperative to unravel the intricate mechanisms by which senescence influences cancer treatment responses and the likelihood of disease recurrence post-therapy. Understanding the molecular signaling pathways regulating therapy-induced senescence and exploring innovative strategies for targeting senescent cells holds promise for optimizing cancer therapy outcomes and enhancing patient resilience to treatments. This evolving landscape opens avenues for tailored and effective interventions, marking a critical stride toward personalized cancer therapeutics.

### 4.2. Senescence as a Barrier to Tumor Progression

Cellular senescence emerges as a formidable barrier in cancer biology, exerting critical tumor suppressive effects that impede cancer cell proliferation and thwart malignant transformation. Senescence, recognized as a tumor suppressive process, orchestrates a cascade of protective mechanisms inhibiting cancer cell proliferation and curtailing the progression from pre-malignant to malignant states. Triggers such as DNA damage, telomere shortening, and oncogene activation prompt senescence, inducing stable growth arrest in damaged cells [79, 80]. This is a robust defense mechanism against tumorigenesis early in life, underscoring senescence as a crucial guardian at the cellular level.

The tumor suppressive effects of senescence extend into the realm of cancer therapies, where it plays a crucial effector role in various anti-cancer modalities, including chemotherapy, radiotherapy, and endocrine therapies. These treatments induce senescence in cancer cells, leading to cytostatic effects that effectively halt cell proliferation. Senescent cells, in turn, release bioactive molecules via the SASP, potentially stimulating an immune response against tumors [81, 82]. For example, chemotherapy-induced senescence has been observed in breast cancer cells, contributing to the treatment's efficacy by impeding the uncontrolled growth of cancer cells [83].

While senescence induced by cancer therapies initially contributes to treatment efficacy, concerns linger regarding its potential impact on patient resilience to treatments and the specter of disease recurrence post-therapy [44]. The pro-aging impacts of senescent cells released through SASP may pose challenges by promoting tumor recurrence or progression after therapy completion [84]. For instance, the SASP-mediated release of inflammatory cytokines and growth factors may create a microenvironment conducive to cancer cell survival and expansion [85].

The emergence of senotherapies presents a promising avenue for enhancing the efficacy of cancer therapies by specifically targeting senescent cells [86]. Senolytic drugs selectively eliminate these cells, while senostatic drugs inhibit their function. Noteworthy compounds like quercetin, navitoclax, and fisetin are actively under investigation for their potential role in interfering with the pro-aging impacts of senescent cells, thereby improving treatment outcomes [8, 36]. This innovative approach promises to refine cancer therapy strategies by mitigating the potential drawbacks of therapy-induced senescence.

Therefore, the intricate balance of senescence as a barrier to tumor progression unveils a nuanced relationship that influences cancer development and treatment responses. From its fundamental role as a tumor suppressive process to its implications in therapy-induced senescence, the multifaceted nature of senescence offers both challenges and opportunities for refining cancer therapies and understanding their long-term impact on patient outcomes.

### 4.3. Challenges in Harnessing Senescence for Therapeutic Benefit

Navigating the potential therapeutic benefits of senescence in the context of cancer treatment presents a complex challenge due to the dual nature of this cellular stress response mechanism. Traditionally viewed as a protective barrier against cancer, senescence induces stable growth arrest in damaged cells, inhibiting their proliferation. However, recent discoveries have introduced a more nuanced perspective, revealing that senescent cells, particularly through the SASP, may paradoxically contribute to oncogenesis and tumor aggressiveness.

The therapeutic implications of senescence are intricately linked to various anticancer modalities, including chemotherapy, radiotherapy, and endocrine therapies [39]. While these treatments induce senescence in cancer cells, effectively halting their proliferation, the pro-aging impacts of senescent cells released through SASP may pose challenges [1]. This phenomenon can reduce patient resilience to treatments and facilitate disease recurrence post-therapy, raising important considerations in managing senescence in cancer therapy [87].

Addressing the complexities associated with senescence in cancer therapy necessitates the development of innovative strategies, and senotherapies emerge as promising avenues [88]. These therapeutic approaches aim to target senescent cells for therapeutic benefit, encompassing senolytic drugs that selectively eliminate these cells and senostatic drugs that inhibit their function [1]. Examples such as quercetin, navitoclax, and fisetin are actively under investigation for their potential role in enhancing treatment outcomes by mitigating the pro-aging effects of senescent cells [67, 87].

The impact of senescence on treatment efficacy further underscores the need for a delicate balance in leveraging its potential therapeutic benefits. The dual role of senescence, acting both as a protective mechanism against cancer and a potential contributor to tumor progression, emphasizes the intricate nature of this interplay. Understanding the molecular signaling pathways regulating senescence induction becomes paramount, as does exploring innovative strategies to modulate its effects. For instance, comprehending how SASP components, including interleukins and growth factors, influence the tumor microenvironment can inform strategies to optimize treatment efficacy and patient outcomes in cancer therapy.

Therefore, while senescence holds promise as a potential therapeutic tool in cancer treatment, the challenges associated with its dual nature necessitate a nuanced and comprehensive approach. Advances in senotherapies, coupled with an in-depth understanding of the molecular intricacies governing senescence induction, are pivotal for overcoming these challenges and optimizing the therapeutic benefits of senescence in the complex landscape of cancer therapy.

### 4.4. Opportunities for Senescence-Targeted Cancer Therapies

Exploring senescence as a focal point in targeted cancer therapies unveils promising opportunities to harness its intrinsic tumor-suppressive effects. One avenue of exploration lies in Therapy-Induced Senescence (TIS), an approach that capitalizes on established cancer treatments like chemotherapy and radiation to induce senescence in tumor cells [89, 90]. TIS refers to a phenomenon where cells undergo senescence due to exposure to certain therapeutic agents or treatments. These treatments can include chemotherapy, radiation therapy, targeted therapy, or immunotherapy for cancer or other diseases. This strategy halts cancer cell proliferation and serves as a platform to identify novel targets, biomarkers, and senotherapeutics. By leveraging TIS, researchers aim to enhance treatment efficacy while minimizing toxicities associated with conventional anticancer therapies.

Understanding the intricate molecular pathways governing senescence in cancer cells is pivotal for identifying therapeutic targets. Researchers delve into these pathways to uncover insights that guide the development of senescence-targeted therapies [91, 92]. This approach can revolutionize cancer treatment through a "one-two punch" strategy. This involves employing agents that induce tumor cell senescence followed by selectively targeting senescent cells, enhancing the precision and effectiveness of the therapeutic intervention. For instance, elucidating the signaling pathways involved in senescence induction may pave the way for developing targeted drugs that selectively exploit these pathways to induce senescence in cancer cells (93).

However, the path toward effective senescence-targeted therapies is challenging [93, 94]. Senolytic therapies, representing a novel approach, focus on selectively eliminating senescent cells while preserving the beneficial effects of senescence [95, 96]. By doing so, senolytic drugs have the potential to enhance treatment responses and mitigate therapy-related side effects. Integrating senolytic therapies into aggressive anticancer regimens may open new avenues for improving patient outcomes in cancer therapy. For instance, drugs like navitoclax have demonstrated senolytic effects by selectively inducing apoptosis in senescent cells, offering a glimpse into the potential of targeted elimination in cancer therapeutics [97].

Characterizing senescent cell heterogeneity based on cell type, tissue of origin, and the nature of the senescence-inducing affront is paramount for unraveling their role in tumorigenesis [98]. This characterization is pivotal for developing targeted cancer therapies, such as the "one-two punch" strategy, which aims to exploit the diverse properties of senescent cells for therapeutic benefit. For example, understanding how senescent cells differ in response to distinct anticancer treatments may guide the development of tailored therapies that capitalize on these variations for enhanced treatment outcomes [34]. Therefore, the exploration of senescence as a target in cancer therapies holds immense promises, with avenues like Therapy-Induced Senescence, molecular pathway elucidation, senolytic therapies, and comprehensive characterization of senescent cell heterogeneity offering opportunities to revolutionize cancer treatment strategies. Overcoming challenges and addressing knowledge gaps in this evolving field are crucial for advancing senescence-targeted therapies and improving outcomes for cancer patients (Table 2).

**Table 2:** Role of Senescence in Cancer Therapies and Senotherapeutic Approaches.

*Therapy Type*	*Induction of Senescence*	*Role of Senescence*	*Senotherapeutic Approach*
***Chemotherapy***	Yes	Halts cancer cell proliferation; enhances immune responses	Senolytic drugs like quercetin, navitoclax, and fisetin are investigated for selective elimination of senescent cells
***Radiotherapy***	Yes	Triggers DNA damage response leading to senescence	Senolytic drugs targeting senescent cells may enhance treatment outcomes
***Endocrine Therapies***	Yes	Induces growth arrest in hormone-sensitive cancers	Senolytic agents disrupt multiple pathways associated with senescence, potentially improving treatment efficacy
***Senotherapeutic Drugs***	N/A	Target senescent cells or inhibit their function	Compounds like quercetin, navitoclax, and fisetin show promise in eliminating senescent cells, potentially enhancing health and lifespan
***Combination Therapies***	N/A	Exploits synergistic effects of senolytic drugs with standard therapies	Combinations of senolytic agents with conventional treatments aim to improve treatment outcomes by mitigating pro-aging impacts of senescent cells

## 5. Cellular Aging Microenvironment and Tumor Progression

### 5.1. SASP and Tumor Microenvironment

Cellular senescence, marked by irreversible cell cycle arrest and its associated feature, the SASP, intricately shape the tumor microenvironment, exerting profound influences on tumor progression. SASP, a hallmark feature of senescent cells, manifests as the secretion of diverse factors, including inflammatory cytokines, chemokines, growth factors, and matrix remodeling proteins [62]. This multifaceted secretory profile endows SASP with dual effects in the tumor microenvironment, exhibiting tumor-suppressive and tumor-promoting properties contingent upon the specific context and cell types involved [99]. Through paracrine signaling, senescent cells can modulate adjacent stromal, immune, and cancer cells, exerting a profound influence on the surrounding tissue environment [37].

Within the tumor microenvironment, senescent cells play a pivotal role in remodeling the neighboring tissues by altering the behavior of proximal cells [37]. SASP-mediated effects can trigger senescence surveillance, suppress tumorigenesis, or inhibit anti-tumor immunity, fostering tumor progression [100]. Understanding the context-dependent nature of SASP is paramount for deciphering its role in shaping the tumor microenvironment and comprehending its implications for cancer development. For instance, the SASP of senescent fibroblasts has been associated with the promotion of tumor invasion and metastasis, underscoring the complex and nuanced effects of SASP in cancer progression [100].

Recent therapeutic advancements have underscored the potential of targeting SASP components or selectively eliminating senescent cells as viable strategies in cancer treatment [101]. Senomorphics, which suppress SASP, and senolytic drugs, inducing senescent cell death, offer promising avenues for modulating the tumor microenvironment. These approaches hold significant potential for enhancing treatment responses and mitigating tumor-promoting effects associated with senescent cells, providing a new dimension to cancer therapy optimization.

The dynamic nature of cellular senescence in cancer emphasizes its context-dependent roles that evolve over time [102]. Senescent cells can reinforce their phenotype through autocrine signaling, transmitting it to neighboring malignant and non-malignant cells in paracrine, resulting in altered tumoral repercussions over time [53]. Recognizing this dynamic interplay is essential for developing nuanced therapeutic strategies that leverage the dual nature of cellular senescence for optimal anticancer interventions. For instance, the persistence of senescent cells in the tumor microenvironment over time may contribute to chronic inflammation and immune suppression, fostering an environment conducive to cancer progression. Therefore, the interrelation between cellular senescence, SASP, and the tumor microenvironment is a multifaceted landscape that significantly impacts tumorigenesis.

### 5.2. Impact of Cellular Aging and Senescent Cells on Cancer Progression

Cellular aging, a multifaceted process encompassing time-dependent changes at the cellular level, emerges as a pivotal player in the initiation and progression of cancer. Cellular aging operates as a robust tumor suppressor mechanism, employing cell cycle arrest through processes such as oncogene-induced senescence [103]. This phenomenon serves as a natural barrier against neoplastic cell growth, emphasizing the intricate interplay between cellular aging and the complicated landscape of cancer development [103]. Additionally, the absence of telomerase activity in normal tissues, leading to telomere dysfunction, acts as an anti-cancer mechanism by curbing indefinite cell expansion and thwarting the development of neoplastic clones [104]. Activating oncogene-induced senescence in response to aberrant cellular signaling pathways is a protective mechanism to prevent the uncontrolled proliferation of potentially cancerous cells. For example, oncogene activation or irreparable DNA damage can induce the activation of ataxia telangiectasia mutated (ATM) and checkpoint kinase, leading to the phosphorylation of histone H2AX and p53. This activation triggers the p53-p21 signaling pathway, which is crucial in halting the cell cycle and inducing senescence. Other pathways like NLRP6-NF-κB-p14ARF-MDM2 and miR-203-ITPKA-MDM2 can also activate the p53-p21 signaling cascade. In specific cases, such as breast carcinoma cells with overexpressed oncogenic ERBB2, senescence can be induced independently of p53 through upregulation of p21. Loss of anti-oncogenes like PTEN can also trigger senescence through various signaling pathways involving Akt-mTOR -p53 and p19ARF-MDM2-p53. These mechanisms highlight how oncogene-induced senescence acts as a safeguard mechanism to prevent the progression of potentially cancerous cells by promoting cell cycle arrest and cellular senescence [51, 105].

The SASP introduces a paradoxical dimension to the role of senescence in cancer progression. Traditionally viewed as a protective mechanism against cancer, SASP induces cell cycle arrest and promotes immune surveillance [62]. However, recent studies reveal its dual nature. SASP components can counterintuitively foster cancer stemness and aggressiveness, contributing to tumor progression and relapse [106]. This intricate dual role underscores the complexity of senescence in cancer pathogenesis and emphasizes the need for a nuanced understanding to facilitate effective therapeutic interventions [107]. The secretion of factors like interleukins and growth factors by senescent cells, known as the SASP, can have beneficial and harmful effects [108]. For example, SASP factors can promote tissue repair, embryonic development, and anti-tumorigenic properties like attracting immune cells to clear pre-malignant senescent cells. However, some SASP factors can also lead to chronic inflammation, fibrogenesis, tumorigenesis, and impaired insulin sensitivity. In the context of cancer, the SASP can either aid in cancer progression or induce growth arrest and apoptosis of cancer cells, depending on the specific interactions between senescent cells and cancer cells [63, 109].

The accumulation of senescent cells within the tumor microenvironment presents profound implications for cancer progression. Innovative therapeutic strategies targeting senescent cells or modulating SASP components offer promising avenues for cancer therapy [110]. Senolytic drugs inducing senescent cell death or senomorphics suppressing SASP represent pioneering approaches to manipulate the tumor microenvironment and optimize treatment outcomes [111]. By harnessing the dual effects of senescence, researchers aim to refine therapeutic strategies that leverage the tumour-suppressive properties while mitigating the tumor-promoting effects associated with senescent cells [112]. For instance, drugs like dasatinib and quercetin have shown senolytic effects by selectively eliminating senescent cells, presenting a potential avenue for therapeutic intervention [62]. Dasatinib and quercetin have shown senolytic effects by selectively eliminating senescent cells, offering a potential therapeutic intervention. The mechanism involves the synergistic action of these compounds in targeting and eliminating senescent cells. Dasatinib, a cancer drug that inhibits the Src tyrosine kinase, induces apoptosis in senescent cells by disrupting pro-survival signaling pathways. On the other hand, quercetin acts by inhibiting the anti-apoptotic protein Bcl-xL, promoting cell death in senescent cells. Studies have demonstrated that the combination of dasatinib and quercetin effectively reduces the burden of senescent cells in various tissues, including aged mice and humans with conditions like diabetic kidney disease. This senolytic therapy targets and eliminates senescent cells linked to age-related chronic diseases, offering a promising approach to alleviate age-related pathologies. The selective elimination of these dysfunctional cells by dasatinib and quercetin presents a novel strategy for improving health outcomes and potentially addressing age-related disorders [113, 114].

### 5.3. Modulating Cellular Aging and Senescence to Influence Tumor Behavior

Cellular aging and the presence of senescent cells emerge as pivotal factors shaping the tumor microenvironment and influencing tumor behavior. Oncogene-induced senescence is a critical tumor suppressor mechanism, erecting a formidable barrier against cancer development by inducing stable growth arrest in damaged cells [115]. This process is an indispensable anti-cancer mechanism, preventing the rampant proliferation of potentially neoplastic cells. To harness this tumor-suppressive potential, understanding the molecular pathways governing oncogene-induced senescence becomes paramount [116]. Exploring targeted therapies that exploit these pathways offers a promising approach to influencing tumor behavior and inhibiting cancer progression [13]. For instance, drugs like MEK inhibitors have shown efficacy in inducing oncogene-induced senescence in certain cancer types, presenting a potential avenue for therapeutic intervention [8]. MEK inhibitors have demonstrated efficacy in inducing oncogene-induced senescence in certain cancer types, offering a potential avenue for therapeutic intervention. For example, inhibitors of MEK and KRAS kinases have been shown to induce senescence in pancreatic ductal carcinoma cells. The associated SASP promotes angiogenesis, which can influence the tumor microenvironment. MEK inhibitors, like trametinib, target the MEK1/2 pathway, leading to cell cycle arrest and senescence induction in cancer cells. This approach presents a promising strategy for halting the proliferation of cancer cells and potentially improving treatment outcomes in specific cancer types [117, 118].

Telomere shortening, a hallmark of cellular aging, contributes to cellular senescence by triggering a DNA damage response leading to cell cycle arrest [119]. The gradual reduction in telomere length limits the replicative capacity of somatic cells, culminating in cellular senescence [120]. Strategies targeting telomere maintenance mechanisms and interventions preventing telomere dysfunction present potential avenues for modulating cellular aging and influencing tumor behavior in cancer [120]. For example, telomerase-targeting therapies, such as imetelstat, aim to inhibit telomerase activity and impede telomere maintenance, offering a unique approach to modulating cellular aging in cancer treatment. Imetelstat is a potent telomerase inhibitor that targets the catalytic subunit of telomerase, thereby disrupting the enzyme's function in maintaining telomere length. By inhibiting telomerase activity, imetelstat induces telomere shortening in cancer cells, leading to cellular senescence or apoptosis. This approach is particularly effective in cancer therapy as it hampers the unlimited replicative potential of cancer cells by inducing senescence or cell death. The inhibition of telomerase activity with imetelstat represents a promising strategy for targeting cancer cells with high telomerase expression and offers a novel therapeutic avenue for combating cancer progression [121, 122].

The SASP adds a layer of complexity to cancer progression, exhibiting both tumor-suppressive and tumor-promoting effects [15]. Modulating SASP components provides an opportunity to influence the tumor microenvironment and alter tumor behavior. By specifically targeting SASP factors or developing interventions that suppress the pro-tumorigenic aspects of SASP, researchers aim to leverage the beneficial effects of senescence while mitigating its detrimental impact on cancer progression. For instance, antibodies against specific SASP components like IL-6 and IL-8 have shown promise in preclinical studies, highlighting the potential for targeted SASP modulation in cancer therapy [123]. Antibodies targeting specific components of the SASP like IL-6 and IL-8 have shown promise in preclinical studies, indicating the potential for targeted SASP modulation in cancer therapy. For instance, IL-6 and IL-8 are key components of the SASP that play roles in tumor proliferation, invasion, and immunosuppression. Specific neutralizing antibodies against these factors have demonstrated efficacy in inhibiting their functions. For example, targeting IL-6 with a neutralizing monoclonal antibody (Mab-IL-6.8) has been shown to completely abolish JAK/STAT signaling and alleviate symptoms of arthritis in a primate model. Similarly, ABX-IL-8, a humanized monoclonal antibody against IL-8, acts as an antagonist impairing IL-8 signaling and attenuating the growth of certain cancer xenograft models. These examples highlight the potential of using antibodies to target specific SASP components like IL-6 and IL-8 as a strategy to modulate the effects of senescent cells in cancer therapy [124].

Modifying cellular aging and senescence has significant therapeutic implications for cancer treatment. Strategies targeting senescent cells, manipulating SASP components, or preventing telomere dysfunction offer innovative approaches to influence tumor behavior and enhance treatment responses [14, 125]. By comprehending the intricate interplay between cellular aging processes and their impact on cancer initiation and progression, researchers can develop tailored therapeutic interventions that exploit the dual nature of senescence for optimal anticancer outcomes [5, 15]. For instance, combining senolytic drugs, targeting senescent cells, and SASP-modulating agents may represent a comprehensive approach to fine-tuning the tumor microenvironment and improving overall treatment efficacy.

## 6. Senescence Escape and Cancer Aggressiveness

### 6.1. Mechanisms of Senescence Bypass in Aging Cells and Cancer Cells

The phenomenon of senescence escape, wherein cells circumvent the senescent state, emerges as a critical determinant in the aggressiveness and progression of cancer. Telomere maintenance mechanisms constitute one of the key avenues through which cells elude senescence. Telomeres, protective structures at the chromosomal ends, play a pivotal role in triggering cellular senescence [119]. In cancer cells, activating telomerase or engaging alternative lengthening telomeres (ALT) mechanisms is a common strategy to counteract telomere shortening-induced senescence. This allows cancer cells to evade growth arrest and persist in unbridled proliferation [14]. For instance, telomerase activation is frequently observed in cancer cells, ensuring the preservation of telomere length and facilitating continuous cellular division. Understanding the intricate interplay between these telomere maintenance mechanisms and senescence escape is crucial for unraveling the driving forces behind cancer aggressiveness [14, 126].

The activation of oncogenes represents another influential mechanism that drives senescence escape. Oncogene activation can induce replicative stress, damaging DNA and subsequent cellular senescence [115, 127]. However, cancer cells can acquire additional mutations that suppress the senescence response triggered by oncogene activation. Cancer cells gain a proliferative advantage by bypassing this oncogene-induced senescence and exhibit increased aggressiveness, thereby contributing to tumor progression [13, 115]. For instance, mutations in the TP53 gene are commonly associated with the evasion of oncogene-induced senescence, allowing cancer cells to override growth inhibitory signals [13, 128].

The SASP also contributes to promoting senescence escape and cancer aggressiveness. SASP components secreted by senescent cells create a pro-inflammatory microenvironment that fosters tumor growth and invasiveness. Cancer cells may exploit these inflammatory signals to enhance their survival and metastatic potential, evading the senescent cells' growth-inhibitory effects. Targeting SASP components represents a potential strategy to disrupt this pro-tumorigenic signaling cascade and inhibit senescence escape in cancer cells. For example, drugs that interfere with specific SASP factors, such as IL-6 inhibitors, have shown promise in preclinical studies, highlighting the potential for disrupting the pro-tumorigenic effects associated with SASP [15, 125].

Understanding the intricate mechanisms of senescence escape in aging cells and cancer cells holds significant therapeutic implications for cancer treatment. Targeting pathways involved in telomere maintenance, oncogene activation, or SASP signaling presents opportunities to prevent senescence bypass and inhibit cancer aggressiveness. By developing targeted therapies that specifically disrupt the mechanisms driving senescence escape, researchers aim to curtail tumor progression and improve treatment outcomes for cancer patients. For instance, drugs targeting telomerase, such as imetelstat, are currently being investigated for their potential to inhibit telomere maintenance and impede cancer cell growth.

### 6.2. Senescence Escape and Metastasis

The phenomenon of senescence escape, wherein cells elude the senescent state, is a pivotal factor influencing cancer aggressiveness, particularly contributing to metastasis. Telomere maintenance mechanisms represent a critical avenue through which cells bypass senescence, ultimately influencing metastatic potential [126]. Telomerase activation or ALT empower cells to prevent growth arrest induced by telomere shortening. In the context of cancer, the activation of these mechanisms allows for continuous cell proliferation, fostering tumor aggressiveness and enhancing metastatic potential. For example, the upregulation of telomerase is common in cancer cells, ensuring the maintenance of telomere length and facilitating persistent cellular division [126]. The comprehension of how telomere maintenance contributes to senescence escape is paramount for unraveling the mechanisms that drive cancer progression and metastasis [16].

Oncogene activation is another influential factor tied to metastatic potential. Although activation of oncogenes can initially trigger cellular senescence as a protective mechanism against tumorigenesis, cancer cells can develop strategies to bypass oncogene-induced senescence. This evasion of growth-inhibitory effects endows cancer cells with a proliferative advantage, fueling their capacity to invade surrounding tissues and disseminate to distant sites, thus fostering metastatic behavior [127, 129]. The intricate interplay between oncogene activation, senescence escape, and metastasis underscores the complexity inherent in the process of tumor progression.

Senescent cells within the tumor microenvironment further contribute to metastatic behavior through the secretion of pro-inflammatory factors and extracellular matrix remodeling proteins [15, 125]. This unique secretory profile creates a pro-tumorigenic niche that supports cancer cell invasion, migration, and colonization at distant sites. Targeting SASP components or senescent cells emerges as a potential strategy to disrupt this metastasis-promoting microenvironment, providing an avenue to inhibit cancer aggressiveness [14].

The therapeutic implications of understanding how senescence escape contribute to cancer aggressiveness, and metastasis are profound, particularly in the realm of metastatic cancer treatment. Targeting pathways involved in telomere maintenance, oncogene activation, or SASP signaling offers promising opportunities to prevent senescence bypass and inhibit metastatic spread. The development of tailored therapeutic interventions that disrupt the mechanisms driving senescence escape in cancer cells holds the potential to effectively curb metastatic behavior and improve outcomes for patients grappling with advanced-stage cancers. For instance, ongoing research into telomerase inhibitors aims to leverage telomere maintenance mechanisms as a therapeutic target to impede metastatic progression in various cancer types.

### 6.3. Implications for Cancer Prognosis and Therapy Resistance

The phenomenon of senescence escape, where cells elude the senescent state, carries profound implications for cancer prognosis and the challenge of therapy resistance. Senescence escape refers to the phenomenon where cells bypass or overcome the normal process of cellular senescence, a state of irreversible growth arrest that cells enter in response to various stressors, such as DNA damage or shortened telomeres. Senescence escape in cancer cells emerges as a harbinger of poor prognosis, primarily due to its role in fostering tumor aggressiveness and metastasis [1, 130]. Cells adept at bypassing senescence mechanisms showcase heightened proliferative capacity, invasiveness, and resistance to cell death, collectively contributing to more aggressive tumor behavior [8]. The presence of senescence-escaped cells within the tumor microenvironment correlates with advanced disease stages, heightened metastatic potential, and compromised clinical outcomes [15]. Recognizing the link between senescence escape and cancer prognosis becomes paramount for predicting disease progression and tailoring effective treatment strategies [1, 8]. The presence of senescence-escaped cells in advanced-stage melanomas has been linked to increased metastatic potential, highlighting its importance in predicting disease outcomes. For example, melanoma cells that evade senescence mechanisms can exhibit enhanced aggressiveness and metastatic behavior. These senescence-escaped cells may acquire characteristics that allow them to bypass the growth arrest associated with senescence, leading to uncontrolled proliferation and dissemination, ultimately contributing to disease progression. Understanding the dynamics of these cells is crucial for developing targeted therapies that can effectively address their metastatic behavior and improve treatment outcomes [131].

Senescence escape is a formidable obstacle in cancer therapy, endowing cells with resistance to conventional treatments. Cells proficient in evading senescence mechanisms can withstand the cytotoxic effects of chemotherapy or radiation therapy, leading to treatment failure and subsequent disease recurrence. The acquisition of senescence bypass mechanisms, such as telomere maintenance or oncogene activation, empowers cancer cells to endure therapeutic insults and adapt to treatment-induced stress. Targeting the pathways involved in senescence escape emerges as a promising approach to surmount therapy resistance and enhance treatment outcomes for patients grappling with refractory cancers [14, 132]. The presence of senescence-escaped cells in advanced-stage melanomas has been linked to increased metastatic potential, highlighting its importance in predicting disease outcomes. For example, melanoma cells that evade senescence mechanisms can exhibit enhanced aggressiveness and metastatic behavior. These senescence-escaped cells may acquire characteristics that allow them to bypass the growth arrest associated with senescence, leading to uncontrolled proliferation and dissemination, ultimately contributing to disease progression. Understanding the dynamics of these cells is crucial for developing targeted therapies that can effectively address their metastatic behavior and improve treatment outcomes [131].

The SASP contributes significantly to therapy resistance by creating a pro-inflammatory microenvironment conducive to cancer cell survival and proliferation. SASP components actively promote tumor cell survival, angiogenesis, and immune evasion, fostering resistance to various treatment modalities, including chemotherapy, targeted therapy, or immunotherapy. Strategies aimed at modulating SASP signaling or directly targeting senescent cells present potential avenues to overcome therapy resistance and amplify treatment efficacy in cancer patients. Notably, manipulating SASP components in breast cancer has shown promise in sensitizing resistant cells to chemotherapy, highlighting the therapeutic potential of addressing senescence-related mechanisms [15, 125].

Understanding the implications of senescence escape for cancer prognosis and therapy resistance carries substantial clinical significance in developing novel treatment strategies. Targeting the pathways involved in senescence bypass mechanisms or modulating the effects of SASP on tumor behavior represents a promising avenue to augment the efficacy of existing therapies and overcome resistance mechanisms in cancer. Tailored therapeutic approaches that carefully consider the impact of senescence escape on treatment outcomes hold promise for improving patient survival rates and achieving long-term disease control. For instance, ongoing research into senolytic drugs aims to selectively eliminate senescent cells, potentially mitigating therapy resistance and enhancing treatment responses in various cancers.

## 7. Impact of Cellular Aging and Senescence on Various Cancer Types

### 7.1. Breast Cancer

Senescence is critical in various physiological processes, including development, tissue repair, and aging. However, it also has significant implications for the development and progression of breast cancer. One of the primary mechanisms underlying cellular aging is telomere shortening. Telomeres, repetitive DNA sequences located at the ends of chromosomes, protect the integrity of genetic material during cell division. With each cell division, telomeres progressively shorten. When telomeres become critically short, cells enter a state of replicative senescence, ceasing further division [83]. This mechanism is a natural barrier against uncontrolled cell proliferation, a hallmark of cancer. However, cancer cells can overcome this barrier through telomerase reactivation, bypassing senescence, and continuing unchecked proliferation.

Another aspect of cellular aging impacting breast cancer is DDR. Senescent cells often exhibit persistent DNA damage, activating DDR pathways like p53 and p16INK4a, which promote cell cycle arrest and senescence in response to DNA damage, preventing the propagation of harmful mutations [8, 83]. Dysfunctional DDR signaling can lead to genomic instability and mutation accumulation, predisposing cells to malignant transformation. In breast cancer, defects in DDR pathways are frequent, contributing to tumor initiation and progression.

Senescent cells also exhibit altered secretory profiles, known as the SASP. SASP involves the secretion of pro-inflammatory cytokines, growth factors, and matrix-remodeling enzymes. While initially facilitating tissue repair and immune surveillance, chronic SASP signaling can promote tumor growth and metastasis. In breast cancer, SASP factors foster a tumor-promoting microenvironment, stimulating tumor cell proliferation, angiogenesis, and immune evasion [8, 28].

Interestingly, senescence can exert both tumor-suppressive and tumor-promoting effects in breast cancer, depending on the context. Senescence is a barrier against tumor initiation by halting the proliferation of damaged cells. However, senescent cells can promote tumor progression through paracrine signaling, immune evasion, and tissue remodeling. The balance between these effects is influenced by various factors, including tumor stage, cellular context, and the tumor microenvironment [8, 28]. Hence, cellular aging and senescence play complex roles in breast cancer, influencing tumor initiation, progression, and therapy response. Understanding the interplay between senescence and cancer biology is crucial for developing novel therapeutic strategies targeting senescent cells or modulating senescence-associated pathways to improve breast cancer management.

### 7.2. Lung Cancer

Cellular aging and senescence play pivotal roles in the intricate landscape of lung cancer development and progression. At the core of this relationship lies the process of telomere shortening. Telomeres, protective caps at the ends of chromosomes, progressively erode with each cell division. This erosion acts as a cellular clock, eventually triggering senescence or apoptosis once telomeres reach a critical length. However, cancer cells often exploit mechanisms to circumvent this fate, maintaining telomere length and enabling indefinite proliferation [133].

Moreover, cellular senescence, typically a safeguard against tumorigenesis, can paradoxically fuel cancer progression. Senescent cells adopt a pro-inflammatory phenotype, secreting myriad signaling molecules known as the SASP. In the context of lung cancer, this inflammatory milieu can promote tumor growth and metastasis, fostering a microenvironment conducive to malignancy [134, 135].

DNA damage represents another nexus between aging and lung cancer. Accumulated DNA lesions, stemming from endogenous metabolic processes and exogenous insults like cigarette smoke contribute to genomic instability. While cells possess intricate DNA repair mechanisms, aging compromises these processes, increasing susceptibility to oncogenic mutations and tumor initiation. Furthermore, the interplay between aging and immune surveillance is crucial in lung cancer. Immunosenescence, the age-related decline in immune function, compromises the body's ability to detect and eliminate cancer cells. Senescent cells within the tumor microenvironment further exacerbate immune evasion by fostering an immunosuppressive milieu through SASP-mediated recruitment of regulatory T cells and myeloid-derived suppressor cells [134, 136].

Chronic inflammation induced by environmental insults perpetuates this lung cycle, accelerating cellular aging processes and promoting the growth of premalignant lesions. Lung cancer cells adeptly exploit vulnerabilities in the aging microenvironment, evading senescence checkpoints and immune surveillance to propagate unchecked [136, 137]. Understanding the intricate interplay between cellular aging, senescence, and lung cancer is paramount for developing effective prevention and treatment strategies. By targeting key pathways involved in these processes, such as telomere maintenance, DNA repair mechanisms, and immune modulation, researchers aim to unravel the complexities of lung cancer pathogenesis and devise innovative therapeutic interventions.

### 7.3. Colorectal Cancer

Cellular aging, or senescence, is intricately linked to the onset and progression of colorectal cancer, a prevalent malignancy arising from the colon or rectal lining. A key aspect of cellular aging is telomere shortening, where protective caps at the ends of chromosomes degrade with each cell division. This process often culminates in cellular senescence or programmed cell death. However, in cancer, including colorectal cancer, cells frequently activate telomerase, a mechanism that circumvents this senescence, allowing for uncontrolled proliferation [138, 139].

Moreover, aging cells accumulate DNA damage over time due to various internal and external factors such as reactive oxygen species and environmental toxins. This DNA damage can trigger senescence or programmed cell death pathways. However, cancer cells often develop mechanisms to repair DNA damage or bypass cell cycle checkpoints, enabling their survival and proliferation despite genomic instability [138, 139]. In colorectal cancer, mutations in DNA repair genes like APC contribute significantly to tumor initiation and progression [138].

Another critical aspect of cellular aging is the SASP. In colorectal cancer, SASP components contribute to creating a pro-tumorigenic microenvironment by promoting inflammation, angiogenesis, and tissue remodeling. This microenvironment supports the growth and metastasis of cancer cells, further exacerbating tumor progression [138, 139].

Immune senescence, a consequence of aging, also plays a pivotal role in colorectal cancer. The immune system changes with age, including immunosenescence and chronic low-grade inflammation. These alterations compromise the immune system's ability to recognize and eliminate cancer cells, allowing them to evade immune surveillance and establish tumors. In colorectal cancer, immune senescence contributes to tumor immune evasion and resistance to immunotherapy, limiting treatment effectiveness [45, 140].

Furthermore, aging is associated with extensive epigenetic modifications, such as DNA methylation and histone modifications. These alterations can dysregulate gene expression patterns, promoting oncogenic signaling pathways and silencing tumor suppressor genes in colorectal cancer cells. Additionally, epigenetic changes can drive cellular senescence by altering chromatin structure and transcriptional programs associated with senescence-associated growth arrest [138, 139]. Understanding the intricate interplay between cellular aging, senescence, and colorectal cancer is crucial for developing effective therapeutic strategies. Targeting senescent cells and mitigating age-related risk factors associated with colorectal cancer could lead to innovative treatment approaches, ultimately improving patient outcomes and quality of life.

### 7.4. Prostate Cancer

Cellular aging plays a significant role in the development and progression of prostate cancer. Prostate cancer is the second most common cancer in men worldwide and is strongly associated with aging. As men age, their risk of developing prostate cancer increases, indicating a link between cellular aging processes and the onset of this disease.

In the context of prostate cancer, cellular aging and senescence can have both protective and detrimental effects. On one hand, senescence acts as a tumor-suppressive mechanism by preventing the uncontrolled growth of damaged cells. When cells accumulate DNA damage or mutations, they can undergo senescence to avoid becoming cancerous. This prevents the proliferation of potentially harmful cells and serves as a barrier to tumor development [141, 142]. However, senescent cells also secrete various factors collectively known as the SASP. The SASP includes pro-inflammatory cytokines, growth factors, and matrix metalloproteinases, among other molecules. While the SASP can initially contribute to the clearance of damaged cells and tissue repair, prolonged secretion of these factors can promote chronic inflammation and tissue dysfunction, creating a microenvironment conducive to tumor growth and progression [28, 142].

In the prostate gland, the effects of cellular aging and senescence on prostate cancer are multifaceted. With advancing age, the prostate undergoes structural and functional changes, including alterations in hormone levels, inflammation, and accumulation of DNA damage. These age-related changes can disrupt the balance between cell proliferation and senescence, tipping the scales towards oncogenic transformation [141, 142]. Additionally, the prostate microenvironment undergoes significant remodeling with age, characterized by increased inflammation and tissue fibrosis. These changes create fertile ground for the development and progression of prostate cancer. Senescent cells contribute to this microenvironmental remodeling through the secretion of pro-inflammatory cytokines and matrix-degrading enzymes, further fueling tumor growth and invasion [28, 142].

Furthermore, studies have shown that senescent cells can evade immune surveillance, allowing them to persist within the prostate tissue and contribute to the chronic inflammatory milieu associated with prostate cancer. This immune evasion facilitates tumor progression and metastasis, leading to more aggressive forms of the disease [141, 143]. Therefore, cellular aging and senescence have a profound impact on prostate cancer development and progression. While senescence initially serves as a protective mechanism against oncogenic transformation, the chronic inflammation and tissue remodeling associated with senescent cells creates an environment conducive to tumor growth and metastasis. Understanding the interplay between cellular aging processes and prostate cancer biology is crucial for the development of effective diagnostic and therapeutic strategies targeting this prevalent disease.

### 7.5. Gastric Cancer

Gastric cancer emerges from the uncontrolled growth of cells in the stomach lining, influenced by factors such as Helicobacter pylori infection, chronic inflammation, diet, and genetics. Within this context, cellular aging and senescence play intricate roles, impacting the disease through multiple mechanisms [144, 145].

Telomere shortening, a hallmark of aging, is observed in gastric cancer cells, leading to genomic instability and uncontrolled proliferation. Senescent cells secrete a range of molecules known as the SASP. In gastric cancer, SASP components foster chronic inflammation, angiogenesis, and tumor cell invasion. Furthermore, Oncogene-induced senescence (OIS) acts as a tumor-suppressive mechanism to halt the proliferation of potentially cancerous cells. However, prolonged activation of oncogenes can lead to senescence bypassing mechanisms, enabling cells to evade senescence and continue proliferating, contributing to gastric cancer development [1, 144].

Immune surveillance, crucial for cancer prevention, is altered in cellular senescence. Senescent cells may evade immune detection by upregulating immune checkpoint molecules like PD-L1, hindering the anti-tumor immune response and promoting gastric cancer progression [1, 146]. Moreover, epigenetic alterations, including changes in chromatin structure and DNA methylation patterns, occur in senescent cells, leading to aberrant gene expression profiles that promote gastric cancer development and metastasis [144, 145].

Understanding the interplay between cellular aging, senescence, and gastric cancer is critical for identifying new therapeutic targets. Strategies targeting senescent cells or modulating the tumor microenvironment hold promise for inhibiting tumor growth and improving outcomes for patients with gastric cancer.

### 7.6. Other Cancer Types

Cellular senescence also plays an important role in other types of cancers. For example, in skin cancer, including melanoma and non-melanoma types, cellular senescence induced by factors like UV radiation is a protective mechanism by triggering the permanent growth arrest of damaged melanocytes and keratinocytes. However, senescent cells in the skin microenvironment can also foster tumor progression by secreting factors that enhance tumor cell proliferation, angiogenesis, and immune evasion [147, 148].

Pancreatic cancer presents another scenario where senescence induction by oncogenic stress initially inhibits tumor growth by arresting the proliferation of pre-neoplastic cells. Nonetheless, persistent senescent cells in the tumor microenvironment can promote inflammation and fibrosis, creating a supportive niche for tumor progression, metastasis, and resistance to therapy [36].

Brain cancer, particularly gliomas, exhibits dual effects of cellular senescence depending on tumor stage and genetic context. While senescence induction may initially suppress tumor growth by triggering cell cycle arrest, it can also contribute to tumor recurrence and therapy resistance by promoting the survival and proliferation of senescent tumor cells and activating pro-tumorigenic signaling pathways [149, 150].

Liver cancer, such as hepatocellular carcinoma (HCC), demonstrates a similar pattern, where senescence induced by chronic liver injury and hepatitis viruses can initially eliminate damaged hepatocytes, thus protecting against HCC initiation. However, persistent senescent cells in the liver microenvironment can fuel inflammation, fibrosis, and compensatory proliferation, ultimately promoting HCC development and progression [151, 152].

**Figure 4.**
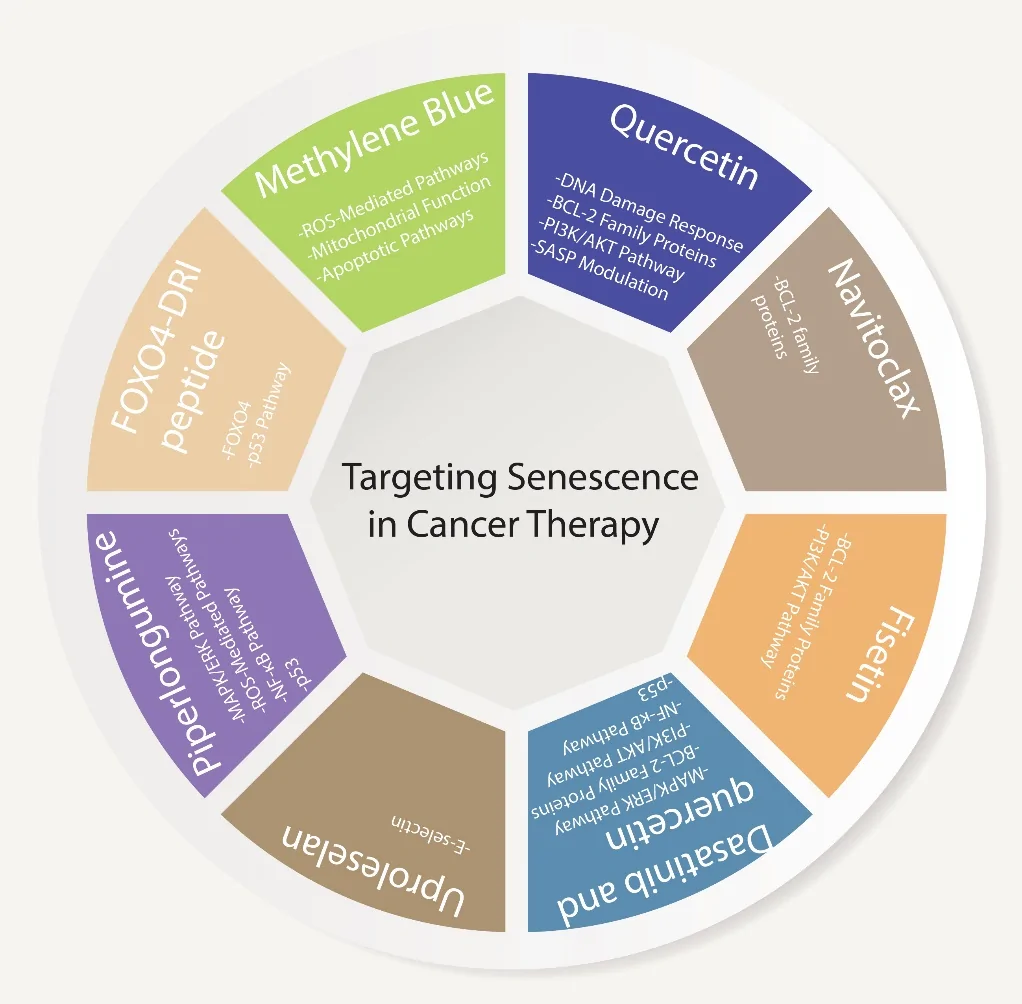
Emerging Senolytic Drugs Targeting Senescence in Cancer Therapy: Overview of Potential Therapeutic Agents and Their Mechanisms of Action.

Bladder cancer and renal cell carcinoma (RCC) also exhibit diverse responses to cellular senescence. In bladder cancer, senescent cells may initially inhibit tumor growth by triggering cell cycle arrest and immune-mediated clearance. However, senescent cells' secretion of SASP factors can promote tumor progression, invasion, and resistance to chemotherapy and immunotherapy. Similarly, in RCC, senescence induction can halt the proliferation of malignant cells. Still, it may also modulate the immune response, promote angiogenesis, and facilitate the establishment of a pre-metastatic niche, ultimately contributing to tumor progression [153, 154].

In summary, the impact of cellular aging and senescence on cancer varies widely across different types of tumors. Understanding these complexities is crucial for developing targeted therapeutic approaches that exploit senescence-associated pathways in cancer treatment.

## 8. Drugs to Target Senescence in Cancer

The field of oncology is witnessing a growing interest in developing pharmaceutical agents that can selectively eliminate senescent cells within tumors, a concept known as "Drugs to Target Senescence in Cancer." Although no longer dividing, Senescent cells remain metabolically active and often contribute to tumor growth and therapy resistance. Compounds like quercetin, navitoclax, and fisetin have shown senolytic properties by inducing selective death in senescent cells. These drugs act on various pathways linked to senescence, including BCL-2 family proteins, the FOXO4-p53 interaction, and cell adhesion molecules such as E-selectin (Fig. 4). While preclinical studies have shown promising results, further research is essential to assess the safety and effectiveness of these drugs in clinical settings.

### 8.1. Quercetin

Quercetin, a flavonoid compound, demonstrates significant promise as a senolytic agent, particularly in cancer settings. This natural compound can selectively target and eliminate senescent cells by interfering with various pathways linked to senescence. Quercetin offers a focused strategy to impede cancer progression by disrupting these pathways. Studies have highlighted the effectiveness of quercetin derivatives, such as QD3, in sensitizing senescent breast cancer cells induced by chemotherapy, showcasing a novel approach to combat cancer by combining pro-senescence and senolytic activities [155, 156]. The pleiotropic effects of quercetin on cellular senescence underscore its potential as a valuable tool in anti-cancer strategies [97]. Additionally, research has explored the synergistic effects of quercetin with other compounds like dasatinib to enhance its senolytic action against cancer cells [157]. These findings collectively emphasize the promising role of quercetin as a targeted senolytic agent in cancer therapy, offering new avenues for combating cancer progression through the elimination of senescent cells.

### 8.2. Navitoclax

Navitoclax (ABT-263), a precision drug, is designed to target BCL-2 family proteins to initiate apoptosis selectively in senescent cells in cancerous tissues. By specifically inducing programmed cell death in these senescent cells, Navitoclax aims to reduce the load of senescent cells, potentially improving the effectiveness of treatments for cancer patients. Studies have shown that Navitoclax effectively eliminates senescent cells in various contexts, such as human umbilical vein epithelial cells, human lung fibroblasts, and murine embryonic fibroblasts, highlighting its senolytic activity [158]. This targeted approach to triggering apoptosis in senescent cells underscores the potential of Navitoclax as a valuable tool in combating cancer progression by reducing the presence of these resistant cells within tumors [159, 160]. The ability of Navitoclax to induce apoptosis in specific types of senescent cells offers a promising strategy for enhancing therapeutic outcomes in cancer treatment by addressing the challenges posed by these resilient cells.

### 8.3. Fisetin

Fisetin, a flavonoid compound similar to quercetin, is recognized for its potent senolytic properties, especially in cancer [161]. This natural compound stands out for its ability to induce apoptosis in senescent cells by modulating diverse pathways linked to senescence, showcasing cell-type-specific effects. Fisetin's selective approach in targeting senescent cells highlights its potential as a valuable asset in cancer therapy by effectively eliminating these resistant cells within tumors. Studies have emphasized fisetin's efficacy in inhibiting cell migration, suppressing metastases, and enhancing the effects of chemotherapy across various cancer types, underlining its multifaceted impact on cancer cells [36, 162]. The distinct senolytic activity of fisetin, coupled with its ability to target specific pathways associated with senescence, positions it as a promising candidate for combatting cancer progression through the elimination of senescent cells, offering new avenues for innovative cancer treatments [163, 164].

### 8.4. Dasatinib

Dasatinib, recognized primarily as a tyrosine kinase inhibitor, has exhibited significant senolytic effects, especially in cancer settings, when used in combination with quercetin. This synergistic combination has shown potent senolytic properties, effectively targeting and eliminating age-related senescent cells within cancerous tissues. The selective removal of these senescent cells holds promise for enhancing the efficacy of cancer treatments and potentially extending both patient health and lifespan. Studies have highlighted the effectiveness of dasatinib and quercetin in eliminating senescent cells, emphasizing their potential in anti-cancer strategies by specifically targeting these resistant cells within tumors [157]. This targeted approach to senolysis offers a novel avenue for improving cancer treatment outcomes by addressing the challenges posed by senescent cells within the tumor microenvironment. The combination of dasatinib and quercetin represents a promising strategy for combating cancer progression through the selective elimination of these age-related senescent cells, showcasing the potential for enhancing both the quality of life and longevity of cancer patients [36].

### 8.5. Uproleselan

Uproleselan (GMI-1271) is currently being studied for its senolytic capabilities, specifically emphasising acute myeloid leukemia (AML). This investigational drug targets E-selectin, a vital cell adhesion molecule involved in the interaction between leukemic cells and bone marrow endothelial cells, aiming to disrupt the leukemic microenvironment. By disrupting this interaction, uproleselan shows promise in facilitating the removal of senescent cells within the context of AML, thereby potentially enhancing treatment outcomes for individuals battling cancer. Clinical studies have demonstrated that uproleselan enhances chemotherapy response, improves survival rates, and decreases chemotherapy toxicity in vivo, making it a promising candidate for improving therapeutic strategies in AML patients [165, 166]. The ability of uproleselan to target E-selectin and alter the leukemic microenvironment underscores its potential as a valuable tool in combating AML by promoting the clearance of senescent cells and enhancing the efficacy of existing treatments.

### 8.6. Piperlongumine

Emerging research suggests that piperlongumine demonstrates senolytic effects, potentially by selectively inducing apoptosis in senescent cells, particularly within cancer contexts. This natural compound has shown promising results in targeting and eliminating senescent cells, highlighting its potential for precise intervention in cancer therapy. While piperlongumine's ability to induce apoptosis in senescent cells is promising, further investigations are needed to comprehensively elucidate its mechanism of action and explore its applications in cancer treatment. This compound holds significant promise for offering targeted and effective strategies for combating cancer by addressing the challenges posed by senescent cells within tumors [167, 168]. The selective cytotoxicity of piperlongumine against cancer cells and senescent cells, coupled with its safety profile in non-senescent and non-cancerous cells, underscores its potential as a valuable candidate for further development as a senolytic agent in cancer therapy.

### 8.7. FOXO4-DRI peptide

The FOXO4-DRI peptide demonstrates significant promise in selectively targeting senescent cells for clearance, especially in the field of cancer research. By disrupting the interaction between FOXO4 and p53, this peptide facilitates apoptosis specifically in senescent cells, providing a focused strategy to counteract the aging effects of senescence within cancerous tissues. Research has shown that FOXO4-DRI effectively induces apoptosis in senescent cells by interfering with the FOXO4-p53 complex [169], eliminating these resistant cells within tumors [170, 171]. This targeted approach to promoting apoptosis in senescent cells highlights the potential of FOXO4-DRI as a valuable tool in cancer therapy by addressing the challenges posed by these aging cells within the tumor microenvironment [172]. The ability of FOXO4-DRI to selectively target senescent cells for clearance underscores its promise for enhancing treatment outcomes and potentially extending patient health by mitigating the pro-aging effects associated with senescence in cancer contexts [173].

### 8.8. Methylene Blue

Methylene Blue, traditionally known for its applications as dye and treating methemoglobinemia, has demonstrated senolytic effects in preclinical studies focused on cancer. This compound has shown the ability to selectively eliminate senescent cells and improve healthspan in animal models, indicating its potential for precise intervention in cancer therapy. Research has highlighted the senolytic properties of Methylene Blue, emphasizing its role in targeting and eliminating senescent cells within tumors, thereby offering a promising approach to enhancing cancer treatment outcomes [174, 175]. The selective eradication of senescent cells by Methylene Blue underscores its potential as a valuable tool in cancer therapy by addressing the challenges posed by these aging cells within the tumor microenvironment [176]. The findings from preclinical studies support the notion that Methylene Blue holds promise for targeted intervention in cancer treatment by effectively targeting and eliminating senescent cells, thereby potentially improving therapeutic strategies and patient outcomes.

## 9. Emerging Research and Future Directions

The ever-evolving landscape of research on cellular aging, senescence, and their implications in cancer initiation and progression is paving the way for a deeper understanding of the intricate interplay between these processes.

Cutting-edge research endeavors to integrate various cellular aging processes, including senescence, apoptosis, and autophagy, to unravel their collective impact on cancer development [177]. Autophagy can clear harmful cellular species associated with senescence and promote stress coping in cancer cells. Studies suggest that integrating senescence with apoptosis pathways may offer synergistic effects in eliminating cancer cells. The relationship between autophagy and senescence is complex in cancer, with reports suggesting both tumor-suppressive and tumor-promoting roles for autophagy. Autophagy can regulate cellular quality control in both senescent and normal cells, limiting tumorigenesis [178]. Prolonged senescence can lead to cancer development, emphasizing the importance of understanding the balance between beneficial and maladaptive senescence pathways. The impairment of autophagy contributes to immunosenescence, leading to chronic inflammation and age-related diseases. The mechanisms governing the balance between pro-tumorigenic and anti-tumorigenic functions of senescence remain unclear, highlighting the complexity of cellular aging processes in cancer research [179]. By deciphering the crosstalk between these pathways and understanding their dynamic regulation in cancer cells, researchers aim to identify novel therapeutic targets that exploit vulnerabilities arising from dysregulated cellular aging mechanisms. For instance, ongoing studies explore how integrating senescence with apoptosis pathways may offer synergistic effects in eliminating cancer cells, potentially leading to more effective treatment strategies. Understanding how these processes intersect and influence cellular fate decisions is crucial for developing comprehensive strategies to modulate cancer progression.

Recent studies emphasize the sensitivity of the cellular senescence program to physical differences within the microenvironment. Investigating how mechanical cues, extracellular matrix stiffness, and spatial organization influence senescence induction and escape provides valuable insights into the role of the tumor microenvironment in shaping cellular fate [15]. For example, research has demonstrated that alterations in extracellular matrix stiffness can influence the expression of senescence markers in cancer cells, suggesting a role for physical factors in regulating senescence dynamics. By considering the impact of physical factors on senescence, researchers can uncover novel mechanisms that drive cancer aggressiveness and metastasis, potentially leading to innovative therapeutic interventions.

In the realm of cancer treatment, the phenomenon of therapeutic resistance exhibited by senescent cancer cells presents a formidable obstacle. These cells often resist conventional therapies like chemotherapy and radiation due to altered signaling pathways and diminished proliferative capacity. Consequently, they become refractory to treatments primarily targeting actively dividing cells, necessitating novel therapeutic approaches that effectively eliminate or neutralize senescent cells [180, 181].

Moreover, the relationship between tissue aging, cancer risk, and cellular senescence underscores the profound impact of aging on cancer susceptibility. Senescent cell accumulation in aging tissues creates a pro-inflammatory microenvironment conducive to cancer initiation and progression, thereby highlighting the importance of addressing age-related factors in cancer prevention and treatment strategies [8, 182].

However, amidst these challenges lie potential opportunities for improved diagnosis and prognosis by identifying senescence-associated biomarkers. Markers such as senescence-associated beta-galactosidase (SA-β-gal) activity and senescence-associated heterochromatin foci (SAHF) hold promises for diagnosing cancer and predicting patient outcomes. Their detection in tumor biopsies could enable clinicians to stratify patients more effectively, facilitating the development of tailored treatment approaches targeting individual tumors' specific molecular characteristics [182].

The tumor microenvironment (TME) emerges as a pivotal player in the complex landscape of cancer progression, with the dynamic interplay between senescent cells and the TME exerting profound influences on tumor behavior [183, 184]. Notably, senescent stromal cells within the TME have been implicated in promoting tumor growth and metastasis through paracrine signaling mechanisms, underscoring the imperative to target cancer cells and their microenvironment in therapeutic interventions. This recognition emphasizes the necessity of comprehensive treatment strategies that consider the intricate interactions between tumor cells and their surrounding milieu.

In the era of precision medicine, the heterogeneity of senescent cells within tumors presents a formidable challenge that demands personalized treatment approaches. Precision medicine strategies, encompassing targeted therapies and immunotherapies, promise to selectively target senescent cancer cells while minimizing collateral damage to healthy tissues. By leveraging the molecular diversity of senescent cells, clinicians can tailor treatment regimens to the specific characteristics of individual tumors, thereby optimizing therapeutic efficacy and minimizing adverse effects.

As advancements in cancer therapy continue to extend the lifespan of cancer survivors, there arises a new frontier in survivorship care, addressing the long-term consequences of treatment and accelerated aging. With a growing population of long-term survivors confronting the late effects of therapy, strategies to mitigate the adverse effects of cellular senescence have gained traction as emerging areas of research. From lifestyle interventions to pharmacological approaches targeting senescent cells, efforts are underway to alleviate the burden of senescence-associated complications and enhance the quality of life for cancer survivors in the post-treatment phase. Through a multifaceted approach that integrates clinical care, research, and patient advocacy, the journey towards optimizing survivorship outcomes in the face of cellular senescence unfolds as a vital endeavor in the oncological landscape.

**Figure 5.**
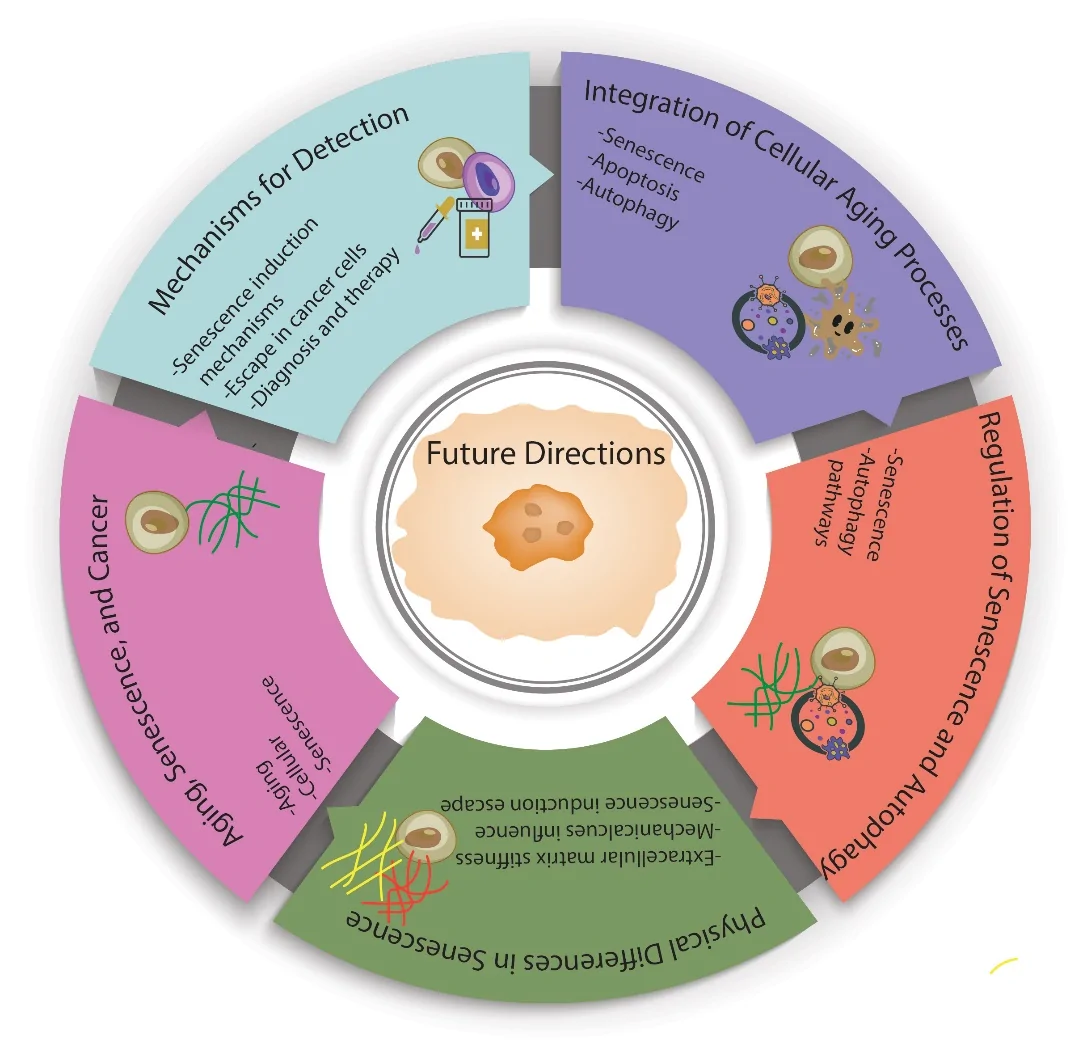
**The figure illustrates the integrated cellular aging processes, emphasizing the roles of senescence, apoptosis, and autophagy in cancer development**. Senescent cells, apoptotic cells, and autophagic processes are depicted within the cellular context, highlighting their collective impact on cancer initiation and progression. Additionally, the figure portrays the complex relationship between senescence and autophagy pathways, demonstrating how autophagy regulates cellular quality control and influences tumorigenesis. The influence of physical factors, such as extracellular matrix stiffness and mechanical cues, on senescence induction and escape is represented, along with alterations in the tumor microenvironment that contribute to cancer aggressiveness and metastasis. Moreover, the figure visualizes the intricate relationship between aging, cellular senescence, and cancer progression, showcasing age-related changes at the cellular level and their implications for tumorigenesis and therapy resistance. Finally, the figure presents molecular mechanisms involved in senescence induction and escape in cancer cells, alongside potential diagnostic tools and precision therapies targeting senescent cells or modulating their secretory phenotype, offering opportunities for early detection and targeted interventions.

Future research directions focus on elucidating the intricate relationship between aging, cellular senescence, and cancer progression. While cellular aging acts as a tumor suppressor mechanism under certain circumstances, it may also enhance cancer development in a context-dependent manner. Investigating how age-related changes at the cellular level contribute to tumorigenesis and therapy resistance provides a foundation for developing personalized treatment approaches that consider the impact of aging on cancer outcomes [185]. For instance, ongoing studies aim to unravel the role of specific age-related alterations in the tumor microenvironment that may influence senescence dynamics and impact cancer behavior. Continued research will yield insights into leveraging cellular aging processes for improved cancer prevention and management.

Advancements in understanding the signaling pathways involved in senescence induction in cancer cells offer opportunities for early detection and targeted interventions. Researchers can develop innovative diagnostic tools and precision therapies that target senescent cells or modulate their secretory phenotype by deciphering the molecular mechanisms that drive senescence escape and promote tumor aggressiveness. For example, identifying specific molecular markers associated with senescence escape may facilitate the development of diagnostic assays for assessing the risk of therapy resistance and disease recurrence. These mechanistic insights pave the way for tailored treatment strategies that address therapy resistance mechanisms associated with senescence escape (Fig. 5) [36, 186].

Therapeutic strategies for addressing cellular aging and senescence in cancer encompass a multifaceted approach aimed at optimizing treatment outcomes. Senolytics, including dasatinib, quercetin, navitoclax, and fisetin, selectively induce apoptosis in senescent cells by targeting specific survival pathways, offering promising avenues for intervention supported by preclinical models and early-phase clinical trials. Immunotherapy presents another compelling strategy, leveraging the immune system to recognize and eliminate senescent cancer cells. Targeting immune checkpoints like PD-1 and CTLA-4 can enhance senescent cell clearance and improve treatment responses. Combination therapies, integrating senolytics with conventional chemotherapy or immunotherapy, offer synergistic effects to overcome treatment resistance and bolster anti-tumor immune responses. Lifestyle modifications such as diet, exercise, and stress reduction are pivotal in modulating cellular senescence and promoting healthy aging. Integrating these interventions into cancer care plans improves treatment tolerability, reduces toxicities, and enhances cancer patients' overall quality of life.

## 10. Conclusion

This comprehensive review explores the intricate interplay between cellular aging, senescence, and their impact on cancer. Beginning with examining cellular aging processes, it uncovers the dual nature of senescence as a tumor suppressor and contributor to cancer development, governed by complex molecular signaling pathways. Insights into senescence's role in cancer therapies highlight its potential as a barrier to tumor progression, while challenges exist in utilizing it for therapeutic benefits. The review emphasizes the importance of the senescence microenvironment, particularly the SASP, offering strategies to manipulate cellular aging and senescence for innovative cancer therapies. Exploration of senescence escape reveals its critical role in cancer aggressiveness, metastasis, and therapy resistance, suggesting targeted interventions. Future directions focus on integrating cellular aging processes and understanding their relationship with physical differences, paving the way for tailored therapeutic strategies and improved cancer outcomes.

## References

[1] Domen A, Deben C, Verswyvel J, Flieswasser T, Prenen H, Peeters M, et al. (2022). Cellular senescence in cancer: clinical detection and prognostic implications. J. Exp. Clin. Cancer Res, 41:360. 10.1186/s13046-022-02555-3 PMC9793681 36575462

[2] Yang J, Liu M, Hong D, Zeng M, Zhang X (2021). The Paradoxical Role of Cellular Senescence in Cancer. Front. cell dev. biol, 9. 10.3389/fcell.2021.722205 PMC8388842 34458273

[3] (2022). The importance of aging in cancer research. Nature Aging, 2:365-366. 10.1038/s43587-022-00231-x 37118075

[4] Campisi J (2013). Aging, Cellular Senescence, and Cancer. Annu. Rev. Physiol, 75:685-705. 10.1146/annurev-physiol-030212-183653 PMC4166529 23140366

[5] Wyld L, Bellantuono I, Tchkonia T, Morgan J, Turner O, Foss F, et al. (2020). Senescence and Cancer: A Review of Clinical Implications of Senescence and Senotherapies. Cancers (Basel), 12. 10.3390/cancers12082134 PMC7464619 32752135

[6] Zinger A, Cho WC, Ben-Yehuda A (2017). Cancer and Aging - the Inflammatory Connection. Aging Dis, 8:611-627. 10.14336/AD.2016.1230 PMC5614325 28966805

[7] Hornsby PJ (2011). Cellular aging and cancer. Crit Rev Oncol Hematol, 79:189-195. 10.1016/j.critrevonc.2010.07.011 PMC3033987 20705476

[8] Schmitt CA, Wang B, Demaria M (2022). Senescence and cancer — role and therapeutic opportunities. Nat. Rev. Clin. Oncol, 19:619-636. 10.1038/s41571-022-00668-4 PMC9428886 36045302

[9] Oda T, Gotoh N, Kasamatsu T, Handa H, Saitoh T, Sasaki N (2023). DNA damage-induced cellular senescence is regulated by 53BP1 accumulation in the nuclear foci and phase separation. Cell Prolif, 56:e13398. 10.1111/cpr.13398 PMC10280147 36642815

[10] Ou HL, Hoffmann R, González-López C, Doherty GJ, Korkola JE, Muñoz-Espín D (2021). Cellular senescence in cancer: from mechanisms to detection. Mol. Oncol, 15:2634-2671. 10.1002/1878-0261.12807 PMC8486596 32981205

[11] Zahid S, Zahid F, Gulzar F, Ali Q, Zahid A, Malik A (2023). Telomere Shortening as Biological Hallmark of Cellular Senescence and Longevity-An Update. Sys Rev Pharm, 14.

[12] Mavrogonatou E, Pratsinis H, Kletsas D (2020). The role of senescence in cancer development. Semin Cancer Biol, 182-191. 10.1016/j.semcancer.2019.06.018 31260734

[13] Zhu H, Blake S, Kusuma FK, Pearson RB, Kang J, Chan KT (2020). Oncogene-induced senescence: From biology to therapy. Mechanisms of Ageing and Development, 187:111229. 10.1016/j.mad.2020.111229 32171687

[14] Yang J, Liu M, Hong D, Zeng M, Zhang X (2021). The Paradoxical Role of Cellular Senescence in Cancer. Front Cell Dev Biol, 9:722205. 10.3389/fcell.2021.722205 PMC8388842 34458273

[15] Takasugi M, Yoshida Y, Hara E, Ohtani N (2023). The role of cellular senescence and SASP in tumour microenvironment. Febs j, 290:1348-1361. 10.1111/febs.16381 35106956

[16] Nassour J, Schmidt TT, Karlseder J (2021). Telomeres and Cancer: Resolving the Paradox. Annu Rev Cancer Biol, 5:59-77. 10.1146/annurev-cancerbio-050420-023410 PMC8442540 34532611

[17] Hemann MT, Narita M (2007). Oncogenes and senescence: breaking down in the fast lane. Genes Dev, 21:1-5. 10.1101/gad.1514207 17210783

[18] Rossiello F, Jurk D, Passos JF, d’Adda di Fagagna F (2022). Telomere dysfunction in ageing and age-related diseases. Nat. Cell Biol, 24:135-147. 10.1038/s41556-022-00842-x PMC8985209 35165420

[19] Victorelli S, Passos JF (2017). Telomeres and Cell Senescence - Size Matters Not. EBioMedicine, 21:14-20. 10.1016/j.ebiom.2017.03.027 PMC5514392 28347656

[20] Park SS, Choi YW, Kim J-H, Kim HS, Park TJ (2021). Senescent tumor cells: an overlooked adversary in the battle against cancer. Exp. Mol. Med, 53:1834-1841. 10.1038/s12276-021-00717-5 PMC8741813 34916607

[21] DeLuca VJ, Saleh T (2023). Insights into the role of senescence in tumor dormancy: mechanisms and applications. Cancer Metastasis Rev, 42:19-35. 10.1007/s10555-023-10082-6 36681750

[22] Wang B, Demaria M (2021). The Quest to Define and Target Cellular Senescence in Cancer. Cancer Res, 81:6087-6089. 10.1158/0008-5472.CAN-21-2032 34911777

[23] Beck J, Turnquist C, Horikawa I, Harris C (2020). Targeting cellular senescence in cancer and aging: roles of p53 and its isoforms. Carcinogenesis, 41:1017-1029. 10.1093/carcin/bgaa071 PMC7422622 32619002

[24] Guerville F, Bourdel-Marchasson I, Déchanet-Merville J, Pellegrin I, Soubeyran P, Appay V, Lemoine M. 2022. Does Inflammation Contribute to Cancer Incidence and Mortality during Aging? A Conceptual Review. In Cancers. Cancers. 10.3390/cancers14071622 PMC8996949 35406394

[25] McHugh D, Gil J (2017). Senescence and aging: Causes, consequences, and therapeutic avenues. JCB, 217:65-77. 10.1083/jcb.201708092 PMC5748990 29114066

[26] Rajaraman R, Guernsey DL, Rajaraman MM, Rajaraman SR (2006). Stem cells, senescence, neosis and self-renewal in cancer. Cancer Cell Int, 6:25. 10.1186/1475-2867-6-25 PMC1664585 17092342

[27] Zaczek A, Kalenik S, Rodacka A (2023). Mechanisms of Senescence in Cancer: Positive and Negative Aspects of Cancer Cells Senescence. Cell Physiol Biochem, 57:478-511. 10.33594/000000671 38112132

[28] Xiao S, Qin D, Hou X, Tian L, Yu Y, Zhang R, et al. (2023). Cellular senescence: a double-edged sword in cancer therapy. Front. oncol, 13. 10.3389/fonc.2023.1189015 PMC10522834 37771436

[29] Lian J, Yue Y, Yu W, Zhang Y (2020). Immunosenescence: a key player in cancer development. J. Hematol. Oncol, 13:151. 10.1186/s13045-020-00986-z PMC7653700 33168037

[30] Prieto Luis I, Baker Darren J (2019). Cellular Senescence and the Immune System in Cancer. Gerontology, 65:505-512. 10.1159/000500683 PMC6703936 31212284

[31] Marin I, Serrano M, Pietrocola F (2023). Recent insights into the crosstalk between senescent cells and CD8 T lymphocytes. npj Aging, 9:8. 10.1038/s41514-023-00105-5 PMC10073090 37015935

[32] Carnero A, Blanco C, Blanco F, Castro ME, Guijarro MV, Fominaya J, et al. (2003). Exploring cellular senescence as a tumor suppressor mechanism. Revista de Oncología, 5:249-265.

[33] Demaria M. 2020. Cellular Senescence and Tumor Promotion. In Geriatric Oncology. Extermann M., editor. Cham: Springer International Publishing. 55-69.

[34] Prasanna PG, Citrin DE, Hildesheim J, Ahmed MM, Venkatachalam S, Riscuta G, et al. (2021). Therapy-Induced Senescence: Opportunities to Improve Anticancer Therapy. JNCI, 113:1285-1298. 10.1093/jnci/djab064 PMC8486333 33792717

[35] Gilioli D, Fusco S, Giannetti K, Gambacorta V, Tavella T, Liberatore C, et al. (2021). Therapy-Induced Senescence As an Anti-Cancer and Immune-Stimulatory Strategy. Blood, 138:4419-4419.

[36] Wang L, Lankhorst L, Bernards R (2022). Exploiting senescence for the treatment of cancer. Nat. Rev. Cancer, 22:340-355. 10.1038/s41568-022-00450-9 35241831

[37] Ovadya Y, Krizhanovsky V (2014). Senescent cells: SASPected drivers of age-related pathologies. Biogerontology, 15:627-642. 10.1007/s10522-014-9529-9 25217383

[38] Liu X, Hoft DF, Peng G (2020). Senescent T cells within suppressive tumor microenvironments: emerging target for tumor immunotherapy. J Clin Invest, 130:1073-1083. 10.1172/JCI133679 PMC7269563 32118585

[39] Schmitt CA, Wang B, Demaria M (2022). Senescence and cancer - role and therapeutic opportunities. Nat Rev Clin Oncol, 19:619-636. 10.1038/s41571-022-00668-4 PMC9428886 36045302

[40] Muñoz-Espín D, Serrano M (2014). Cellular senescence: from physiology to pathology. Nat Rev Mol Cell Biol, 15:482-496. 10.1038/nrm3823 24954210

[41] Demaria M, O'Leary MN, Chang J, Shao L, Liu S, Alimirah F, et al. (2017). Cellular Senescence Promotes Adverse Effects of Chemotherapy and Cancer Relapse. Cancer Discov, 7:165-176. 10.1158/2159-8290.CD-16-0241 PMC5296251 27979832

[42] Coppé JP, Desprez PY, Krtolica A, Campisi J (2010). The senescence-associated secretory phenotype: the dark side of tumor suppression. Annu Rev Pathol, 5:99-118. 10.1146/annurev-pathol-121808-102144 PMC4166495 20078217

[43] Campisi J (2001). Cellular senescence as a tumor-suppressor mechanism. Trends Cell Biol, 11:S27-31. 10.1016/s0962-8924(01)02151-1 11684439

[44] Amaya-Montoya M, Pérez-Londoño A, Guatibonza-García V, Vargas-Villanueva A, Mendivil CO (2020). Cellular Senescence as a Therapeutic Target for Age-Related Diseases: A Review. Adv. Ther, 37:1407-1424. 10.1007/s12325-020-01287-0 PMC7140757 32185730

[45] Kellers F, Fernandez A, Konukiewitz B, Schindeldecker M, Tagscherer KE, Heintz A, et al. (2022). Senescence-Associated Molecules and Tumor-Immune-Interactions as Prognostic Biomarkers in Colorectal Cancer. Front. Med, 9. 10.3389/fmed.2022.865230 PMC9039237 35492321

[46] Liao Z, Yeo HL, Wong SW, Zhao Y. 2021. Cellular Senescence: Mechanisms and Therapeutic Potential. In Biomedicines. 10.3390/biomedicines9121769 PMC8698401 34944585

[47] Herranz N, Gil J (2018). Mechanisms and functions of cellular senescence. J Clin Invest, 128:1238-1246. 10.1172/JCI95148 PMC5873888 29608137

[48] Tan HW, Seen DLT, Xu Y-M, Lau ATY (2023). Cadmium, Cellular Senescence, and Cancer. REV ENVIRON CONTAM T, 261:21.

[49] Mikuła-Pietrasik J, Niklas A, Uruski P, Tykarski A, Książek K (2020). Mechanisms and significance of therapy-induced and spontaneous senescence of cancer cells. CMLS, 77:213-229. 10.1007/s00018-019-03261-8 PMC6970957 31414165

[50] Rossiello F, Herbig U, Longhese MP, Fumagalli M, d’Adda di Fagagna F (2014). Irreparable telomeric DNA damage and persistent DDR signalling as a shared causative mechanism of cellular senescence and ageing. Curr Opin Genet Dev, 26:89-95. 10.1016/j.gde.2014.06.009 PMC4217147 25104620

[51] Huang W, Hickson LJ, Eirin A, Kirkland JL, Lerman LO (2022). Cellular senescence: the good, the bad and the unknown. Nat. Rev. Nephrol, 18:611-627. 10.1038/s41581-022-00601-z PMC9362342 35922662

[52] Aladjem MI. 2014. Establishment of Replicative Immortality in Cancer Cells. In Pathobiology of Human Disease. San Diego: Academic Press. 381-392.

[53] Kumari R, Jat P (2021). Mechanisms of cellular senescence: cell cycle arrest and senescence associated secretory phenotype. Front. cell dev. biol, 9:485. 10.3389/fcell.2021.645593 PMC8039141 33855023

[54] Nikfarjam S, Singh KK (2023). DNA damage response signaling: A common link between cancer and cardiovascular diseases. Cancer Med, 12:4380-4404. 10.1002/cam4.5274 PMC9972122 36156462

[55] Abbadie C, Pluquet O (2020). Unfolded Protein Response (UPR) Controls Major Senescence Hallmarks. TIBS, 45:371-374. 10.1016/j.tibs.2020.02.005 32311331

[56] Pluquet O, Pourtier A, Abbadie C (2015). The unfolded protein response and cellular senescence. A review in the theme: cellular mechanisms of endoplasmic reticulum stress signaling in health and disease. Am J Physiol Cell Physiol, 308:C415-425. 10.1152/ajpcell.00334.2014 25540175

[57] Sabath N, Levy-Adam F, Younis A, Rozales K, Meller A, Hadar S, et al. (2020). Cellular proteostasis decline in human senescence. PNAS, 117:31902-31913. 10.1073/pnas.2018138117 PMC7749315 33257563

[58] Pluquet O, Pourtier A, Abbadie C (2014). The unfolded protein response and cellular senescence. A Review in the Theme: Cellular Mechanisms of Endoplasmic Reticulum Stress Signaling in Health and Disease. Am. J. Physiol. Cell Physiol, 308:C415-C425. 10.1152/ajpcell.00334.2014 25540175

[59] Mühleder S, Fernández-Chacón M, Garcia-Gonzalez I, Benedito R (2021). Endothelial sprouting, proliferation, or senescence: tipping the balance from physiology to pathology. CMLS, 78:1329-1354. 10.1007/s00018-020-03664-y PMC7904752 33078209

[60] Horikawa I, Fujita K, Harris CC (2011). p53 governs telomere regulation feedback too, via TRF2. Aging, 3:26-32. 10.18632/aging.100271 PMC3047136 21266744

[61] Salminen A (2021). Feed-forward regulation between cellular senescence and immunosuppression promotes the aging process and age-related diseases. ARR, 67:101280. 10.1016/j.arr.2021.101280 33581314

[62] Ohtani N (2022). The roles and mechanisms of senescence-associated secretory phenotype (SASP): can it be controlled by senolysis? Inflammation and Regeneration, 42:11. 10.1186/s41232-022-00197-8 PMC8976373 35365245

[63] Ohtani N (2019). Deciphering the mechanism for induction of senescence-associated secretory phenotype (SASP) and its role in ageing and cancer development. [J]. Biochem, 166:289-295. 10.1093/jb/mvz055 31297533

[64] He C, Lv X, Cong H, Hua G, Ma B, Chen X, et al. (2018). Abstract B43: Disruption of the YAP-LATS2 feedback loop switches ovarian cells from YAP-induced senescence to malignant transformation. Clin Cancer Res, 24:B43-B43.

[65] Langhi Prata LGP, Tchkonia T, Kirkland JL (2023). Cell senescence, the senescence-associated secretory phenotype, and cancers. PLoS Biol, 21:e3002326. 10.1371/journal.pbio.3002326 PMC10547493 37733806

[66] 2023. imt.ie: Iresh Medical Times.

[67] Mongiardi MP, Pellegrini M, Pallini R, Levi A, Falchetti ML. 2021. Cancer Response to Therapy-Induced Senescence: A Matter of Dose and Timing. In Cancers. 10.3390/cancers13030484 PMC7865402 33513872

[68] Kallenbach J, Atri Roozbahani G, Heidari Horestani M, Baniahmad A (2022). Distinct mechanisms mediating therapy-induced cellular senescence in prostate cancer. Cell & Bioscience, 12:200. 10.1186/s13578-022-00941-0 PMC9753376 36522745

[69] Saleh T, Bloukh S, Carpenter VJ, Alwohoush E, Bakeer J, Darwish S, et al.2020. Therapy-Induced Senescence: An “Old” Friend Becomes the Enemy. In Cancers. 10.3390/cancers12040822 PMC7226427 32235364

[70] Hwang HV, Tran DT, Rebuffatti MN, Li CS, Knowlton AA (2018). Investigation of quercetin and hyperoside as senolytics in adult human endothelial cells. PLoS One, 13:e0190374. 10.1371/journal.pone.0190374 PMC5760026 29315311

[71] Fatt MP, Tran LM, Vetere G, Storer MA, Simonetta JV, Miller FD, et al. (2022). Restoration of hippocampal neural precursor function by ablation of senescent cells in the aging stem cell niche. Stem Cell Reports, 17:259-275. 10.1016/j.stemcr.2021.12.010 PMC8828532 35063124

[72] Rad AN, Grillari J (2024). Current senolytics: Mode of action, efficacy and limitations, and their future. Mech Ageing Dev, 217:111888. 10.1016/j.mad.2023.111888 38040344

[73] Yousefzadeh MJ, Zhu YI, McGowan SJ, Angelini L, Fuhrmann-Stroissnigg H, Xu M, et al. (2018). Fisetin is a senotherapeutic that extends health and lifespan. EBioMedicine, 36:18-28. 10.1016/j.ebiom.2018.09.015 PMC6197652 30279143

[74] Zhang L, Pitcher LE, Prahalad V, Niedernhofer LJ, Robbins PD (2023). Targeting cellular senescence with senotherapeutics: senolytics and senomorphics. FEBS J, 290:1362-1383. 10.1111/febs.16350 35015337

[75] Zhu M, Meng P, Ling X, Zhou L (2020). Advancements in therapeutic drugs targeting of senescence. TACD, 11:2040622320964125. 10.1177/2040622320964125 PMC7576933 33133476

[76] Shukla R, Pandey V, Vadnere GP, Lodhi S. 2019. Chapter 18 - Role of Flavonoids in Management of Inflammatory Disorders. In Bioactive Food as Dietary Interventions for Arthritis and Related Inflammatory Diseases (Second Edition). Watson R.R., and Preedy V.R., editors: Academic Press. 293-322.

[77] Reyes-Farias M, Carrasco-Pozo C (2019). The Anti-Cancer Effect of Quercetin: Molecular Implications in Cancer Metabolism. Int J Mol Sci, 20. 10.3390/ijms20133177 PMC6651418 31261749

[78] Nor Hisam NS, Ugusman A, Rajab NF, Ahmad MF, Fenech M, Liew SL, Mohamad Anuar NN (2021). Combination Therapy of Navitoclax with Chemotherapeutic Agents in Solid Tumors and Blood Cancer: A Review of Current Evidence. Pharmaceutics, 13. 10.3390/pharmaceutics13091353 PMC8468743 34575429

[79] Chiu F-Y, Kvadas RM, Mheidly Z, Shahbandi A, Jackson JG (2023). Could senescence phenotypes strike the balance to promote tumor dormancy? Cancer Metastasis Rev, 42:143-160. 10.1007/s10555-023-10089-z PMC10710690 36735097

[80] Lucas V, Cavadas C, Aveleira CA (2023). Cellular senescence: from mechanisms to current biomarkers and senotherapies. Pharmacol. Rev, 75:675-713. 10.1124/pharmrev.122.000622 36732079

[81] Özdemir A, Demir YDŞ, Yeşilyurt ZE, Ark M (2023). Senescent cells and SASP in cancer microenvironment: New approaches in cancer therapy. Adv. Protein Chem. Struct. Biol, 133:115-158. 10.1016/bs.apcsb.2022.10.002 36707199

[82] Kim JH, Brown SL, Gordon MN (2023). Radiation-induced senescence: therapeutic opportunities. Radiat. Oncol. J, 18:1-11. 10.1186/s13014-022-02184-2 PMC9837958 36639774

[83] Milczarek M. 2020. The Premature Senescence in Breast Cancer Treatment Strategy. In Cancers. 10.3390/cancers12071815 PMC7408867 32640718

[84] Calcinotto A, Alimonti A (2017). Aging tumour cells to cure cancer: "pro-senescence" therapy for cancer. Swiss Med Wkly, 147:w14367. 10.57187/smw.2017.14367 28102876

[85] Morales-Valencia J, David G (2021). The contribution of physiological and accelerated aging to cancer progression through senescence-induced inflammation. Front. oncol, 11:747822. 10.3389/fonc.2021.747822 PMC8490756 34621683

[86] Qi X, Jiang L, Cao J (2022). Senotherapies: A novel strategy for synergistic anti-tumor therapy. Drug Discov. Today, 27:103365. 10.1016/j.drudis.2022.103365 36115631

[87] He K, Lu L, Jian YP, Xu ZX (2022). Editorial: Induced cell senescence as a therapeutic strategy for cancer treatment. Front Oncol, 12:1104877. 10.3389/fonc.2022.1104877 PMC9768798 36568196

[88] Malayaperumal S, Marotta F, Kumar MM, Somasundaram I, Ayala A, Pinto MM, et al. (2023). The Emerging Role of Senotherapy in Cancer: A Comprehensive Review. Clin Pract, 13:838-852. 10.3390/clinpract13040076 PMC10366900 37489425

[89] Fakhri S, Moradi SZ, DeLiberto LK, Bishayee A (2022). Cellular senescence signaling in cancer: A novel therapeutic target to combat human malignancies. Biochem. Pharmacol, 199:114989. 10.1016/j.bcp.2022.114989 35288153

[90] Roninson IB (2003). Tumor cell senescence in cancer treatment. Cancer Res, 63:2705-2715. 12782571

[91] Ben-Porath I, Weinberg RA (2005). The signals and pathways activating cellular senescence. Int. J. Biochem. Cell Biol, 37:961-976. 10.1016/j.biocel.2004.10.013 15743671

[92] Nasrolahi A, Azizidoost S, Radoszkiewicz K, Najafi S, Ghaedrahmati F, Anbiyaee O, et al. (2023). Signaling pathways governing glioma cancer stem cells behavior. Cell. Signal, 101:110493. 10.1016/j.cellsig.2022.110493 36228964

[93] Paez-Ribes M, González-Gualda E, Doherty GJ, Muñoz-Espín D (2019). Targeting senescent cells in translational medicine. EMBO Mol. Med, 11:e10234. 10.15252/emmm.201810234 PMC6895604 31746100

[94] Ovadya Y, Krizhanovsky V (2018). Strategies targeting cellular senescence. JCI, 128:1247-1254. 10.1172/JCI95149 PMC5873866 29608140

[95] Zhang J-W, Zhang D, Yu B-P (2021). Senescent cells in cancer therapy: Why and how to remove them. Cancer Lett, 520:68-79. 10.1016/j.canlet.2021.07.002 34237406

[96] Myrianthopoulos V, Evangelou K, Vasileiou PVS, Cooks T, Vassilakopoulos TP, Pangalis GA, et al. (2019). Senescence and senotherapeutics: a new field in cancer therapy. Clin. Pharm. Therap, 193:31-49. 10.1016/j.pharmthera.2018.08.006 30121319

[97] Carpenter VJ, Saleh T, Gewirtz DA. 2021. Senolytics for Cancer Therapy: Is All that Glitters Really Gold? In Cancers. 10.3390/cancers13040723 PMC7916462 33578753

[98] Huanyin T, Anke G, Tengjiao Z, Chen W, Ying J, Zhiyong M (2019). Single senescent cell sequencing reveals heterogeneity in senescent cells induced by telomere erosion. Protein&Cell, 10:370-375. 10.1007/s13238-018-0591-y PMC6468032 30421359

[99] Özdemir A, Şimay Demir YD, Yeşilyurt ZE, Ark M (2023). Senescent cells and SASP in cancer microenvironment: New approaches in cancer therapy. Adv. Protein Chem. Struct. Biol, 133:115-158. 10.1016/bs.apcsb.2022.10.002 36707199

[100] Tchkonia T, Zhu Y, van Deursen J, Campisi J, Kirkland JL (2013). Cellular senescence and the senescent secretory phenotype: therapeutic opportunities. J Clin Invest, 123:966-972. 10.1172/JCI64098 PMC3582125 23454759

[101] Xiao S, Qin D, Hou X, Tian L, Yu Y, Zhang R, et al. (2023). Cellular senescence: a double-edged sword in cancer therapy. Front Oncol, 13:1189015. 10.3389/fonc.2023.1189015 PMC10522834 37771436

[102] Lee S, Schmitt CA (2019). The dynamic nature of senescence in cancer. Nat. Cell Biol, 21:94-101. 10.1038/s41556-018-0249-2 30602768

[103] Rattanavirotkul N, Kirschner K, Chandra T (2021). Induction and transmission of oncogene-induced senescence. CMLS, 78:843-852. 10.1007/s00018-020-03638-0 PMC7897614 32936311

[104] Jafri MA, Ansari SA, Alqahtani MH, Shay JW (2016). Roles of telomeres and telomerase in cancer, and advances in telomerase-targeted therapies. Genome Med, 8:69. 10.1186/s13073-016-0324-x PMC4915101 27323951

[105] Reddy JP, Li Y (2011). Oncogene-Induced Senescence and its Role in Tumor Suppression. J. Mammary Gland Biol. Neoplasia, 16:247-256. 10.1007/s10911-011-9221-5 21681694

[106] Saleh T, Tyutynuk-Massey L, Cudjoe EK Jr., Idowu MO, Landry JW, Gewirtz DA (2018). Non-Cell Autonomous Effects of the Senescence-Associated Secretory Phenotype in Cancer Therapy. Front Oncol, 8:164. 10.3389/fonc.2018.00164 PMC5968105 29868482

[107] Chambers CR, Ritchie S, Pereira BA, Timpson P (2021). Overcoming the senescence-associated secretory phenotype (SASP): a complex mechanism of resistance in the treatment of cancer. Mol. Oncol, 15:3242-3255. 10.1002/1878-0261.13042 PMC8637570 34137158

[108] Langhi Prata LGP, Tchkonia T, Kirkland JL (2023). Cell senescence, the senescence-associated secretory phenotype, and cancers. PLoS biol, 21:e3002326. 10.1371/journal.pbio.3002326 PMC10547493 37733806

[109] Banerjee P, Kotla S, Reddy Velatooru L, Abe RJ, Davis EA, Cooke JP, et al. (2021). Senescence-associated secretory phenotype as a hinge between cardiovascular diseases and cancer. Front. cardiovasc. med, 8:763930. 10.3389/fcvm.2021.763930 PMC8563837 34746270

[110] Ruhland MK, Alspach E (2021). Senescence and immunoregulation in the tumor microenvironment. Front. cell dev. biol, 9:754069. 10.3389/fcell.2021.754069 PMC8529213 34692707

[111] Zhao S, Qiao Z, Pfeifer R, Pape H-C, Mao K, Tang H, et al. (2024). Modulation of fracture healing by senescence-associated secretory phenotype (SASP): a narrative review of the current literature. Eur. J. Med. Res, 29:38. 10.1186/s40001-023-01604-7 PMC10775505 38195489

[112] Gabai Y, Assouline B, Ben-Porath I (2023). Senescent stromal cells: roles in the tumor microenvironment. Trends in Cancer, 9:28-41. 10.1016/j.trecan.2022.09.002 36208990

[113] Hickson LJ, Langhi Prata LGP, Bobart SA, Evans TK, Giorgadze N, Hashmi SK, et al. (2019). Senolytics decrease senescent cells in humans: Preliminary report from a clinical trial of Dasatinib plus Quercetin in individuals with diabetic kidney disease. EBioMedicine, 47:446-456. 10.1016/j.ebiom.2019.08.069 PMC6796530 31542391

[114] Saccon TD, Nagpal R, Yadav H, Cavalcante MB, Nunes ADC, Schneider A, et al. (2021). Senolytic Combination of Dasatinib and Quercetin Alleviates Intestinal Senescence and Inflammation and Modulates the Gut Microbiome in Aged Mice. J Gerontol A Biol Sci Med Sci, 76:1895-1905. 10.1093/gerona/glab002 PMC8514064 33406219

[115] Liu XL, Ding J, Meng LH (2018). Oncogene-induced senescence: a double edged sword in cancer. Acta Pharmacol Sin, 39:1553-1558. 10.1038/aps.2017.198 PMC6289471 29620049

[116] Zhu H, Blake S, Kusuma FK, Pearson RB, Kang J, Chan KT (2020). Oncogene-induced senescence: From biology to therapy. Mech Ageing Dev, 187:111229. 10.1016/j.mad.2020.111229 32171687

[117] Mongiardi MP, Pellegrini M, Pallini R, Levi A, Falchetti ML (2021). Cancer Response to Therapy-Induced Senescence: A Matter of Dose and Timing. Cancers (Basel), 13. 10.3390/cancers13030484 PMC7865402 33513872

[118] Yang J, Liu M, Hong D, Zeng M, Zhang X (2021). The paradoxical role of cellular senescence in cancer. Front. cell dev. biol, 9:722205. 10.3389/fcell.2021.722205 PMC8388842 34458273

[119] Ou H-L, Hoffmann R, González-López C, Doherty GJ, Korkola JE, Muñoz-Espín D (2021). Cellular senescence in cancer: from mechanisms to detection. Mol. Oncol, 15:2634-2671. 10.1002/1878-0261.12807 PMC8486596 32981205

[120] Ali JH, Walter M (2023). Combining old and new concepts in targeting telomerase for cancer therapy: transient, immediate, complete and combinatory attack (TICCA). Cancer Cell Int, 23:197. 10.1186/s12935-023-03041-2 PMC10483736 37679807

[121] Shen Z, Wang Y, Wang G, Gu W, Zhao S, Hu X, et al. (2023). Research progress of small-molecule drugs in targeting telomerase in human cancer and aging. Chemico-Biological Interactions, 382:110631. 10.1016/j.cbi.2023.110631 37451664

[122] Fragkiadaki P, Renieri E, Kalliantasi K, Kouvidi E, Apalaki E, Vakonaki E, et al. (2022). Τelomerase inhibitors and activators in aging and cancer: A systematic review. Mol Med Rep, 25:158. 10.3892/mmr.2022.12674 PMC8941523 35266017

[123] Cuollo L, Antonangeli F, Santoni A, Soriani A (2020). The Senescence-Associated Secretory Phenotype (SASP) in the Challenging Future of Cancer Therapy and Age-Related Diseases. Biology (Basel), 9. 10.3390/biology9120485 PMC7767554 33371508

[124] Von Kobbe C (2019). Targeting senescent cells: approaches, opportunities, challenges. Aging (Albany NY), 11:12844. 10.18632/aging.102557 PMC6949083 31789602

[125] Chambers CR, Ritchie S, Pereira BA, Timpson P (2021). Overcoming the senescence-associated secretory phenotype (SASP): a complex mechanism of resistance in the treatment of cancer. Mol Oncol, 15:3242-3255. 10.1002/1878-0261.13042 PMC8637570 34137158

[126] Gao J, Pickett HA (2022). Targeting telomeres: advances in telomere maintenance mechanism-specific cancer therapies. Nat. Rev. Cancer, 22:515-532. 10.1038/s41568-022-00490-1 35790854

[127] Liu X-l, Ding J, Meng L-h (2018). Oncogene-induced senescence: a double edged sword in cancer. Acta Pharmacol. Sin, 39:1553-1558. 10.1038/aps.2017.198 PMC6289471 29620049

[128] Rivlin N, Brosh R, Oren M, Rotter V (2011). Mutations in the p53 Tumor Suppressor Gene: Important Milestones at the Various Steps of Tumorigenesis. Genes Cancer, 2:466-474. 10.1177/1947601911408889 PMC3135636 21779514

[129] Hemann MT, Narita M (2007). Oncogenes and senescence: breaking down in the fast lane. Genes Dev, 21:1-5. 10.1101/gad.1514207 17210783

[130] Pluquet O, Abbadie C, Coqueret O (2019). Connecting cancer relapse with senescence. Cancer Lett, 463:50-58. 10.1016/j.canlet.2019.08.004 31404612

[131] Bennett DC (2016). Genetics of melanoma progression: the rise and fall of cell senescence. PCMR, 29:122-140. 10.1111/pcmr.12422 26386262

[132] Zampetidis CP, Papantonis A, Gorgoulis VG (2022). Escape from senescence: revisiting cancer therapeutic strategies. Mol Cell Oncol, 9:2030158. 10.1080/23723556.2022.2030158 PMC8890391 35252554

[133] Jha SK, De Rubis G, Devkota SR, Zhang Y, Adhikari R, Jha LA, et al. (2024). Cellular senescence in lung cancer: Molecular mechanisms and therapeutic interventions. ARR, 97:102315. 10.1016/j.arr.2024.102315 38679394

[134] Lin W, Wang X, Xu Z, Wang Z, Liu T, Cao Z, et al. (2021). Identification and validation of cellular senescence patterns to predict clinical outcomes and immunotherapeutic responses in lung adenocarcinoma. Cancer Cell Int, 21:652. 10.1186/s12935-021-02358-0 PMC8647370 34872577

[135] Asaithamby A, Shay JW, Minna JD (2022). Cellular senescence and lung cancer prognosis. Transl Lung Cancer Res, 11:1982-1987. 10.21037/tlcr-22-678 PMC9641047 36386455

[136] Hansel C, Jendrossek V, Klein D (2020). Cellular Senescence in the Lung: The Central Role of Senescent Epithelial Cells. Int J Mol Sci, 21. 10.3390/ijms21093279 PMC7247355 32384619

[137] Parikh P, Wicher S, Khandalavala K, Pabelick CM, Britt RD, Prakash YS (2019). Cellular senescence in the lung across the age spectrum. Am. J. Physiol. Lung Cell. Mol. Physiol, 316:L826-L842. 10.1152/ajplung.00424.2018 PMC6589594 30785345

[138] Wu Y, Xie M, Sun JH, Li CC, Dong GH, Zhang QS, Cui PL (2022). Cellular senescence: a promising therapeutic target in colorectal cancer. Future Oncol, 18:3463-3470. 10.2217/fon-2021-0661 36069254

[139] Dong K, Liu J, Zhou W, Zhang G (2022). Exploring the Relationship Between Senescence and Colorectal Cancer in Prognosis, Immunity, and Treatment. Front. genet, 13. 10.3389/fgene.2022.930248 PMC9240351 35783270

[140] Shin JS, Kim T-G, Kim YH, Eom SY, Park SH, Lee DH, et al. (2023). Senescent tumor cells in colorectal cancer are characterized by elevated enzymatic activity of complexes 1 and 2 in oxidative phosphorylation. J Pathol Transl Med, 57:305-314. 10.4132/jptm.2023.10.09 PMC10660360 37926982

[141] Han C, Deng Y, Yang B, Hu P, Hu B, Wang T, et al. (2023). Identification of a novel senescence-associated signature to predict biochemical recurrence and immune microenvironment for prostate cancer. Front. immunol, 14. 10.3389/fimmu.2023.1126902 PMC9986540 36891298

[142] Kallenbach J, Atri Roozbahani G, Heidari Horestani M, Baniahmad A (2022). Distinct mechanisms mediating therapy-induced cellular senescence in prostate cancer. Cell Biosci, 12:200. 10.1186/s13578-022-00941-0 PMC9753376 36522745

[143] Feng D, Li D, Shi X, Xiong Q, Zhang F, Wei Q, Yang L (2022). A gene prognostic index from cellular senescence predicting metastasis and radioresistance for prostate cancer. J. Transl. Med, 20:252. 10.1186/s12967-022-03459-8 PMC9164540 35658892

[144] Liu Y, Zhang Q, Ni W, Ji G, Xu H (2023). A strategy for the treatment of gastrointestinal cancer: Targeting tumor senescent cells. Front Mol Biosci, 10:1139840. 10.3389/fmolb.2023.1139840 PMC10025555 36950520

[145] Takeuchi A, Asano N, Imatani A, Saito M, Jin X, Saito M, et al. (2022). Suppressed Cellular Senescence Mediated by T-box3 in Aged Gastric Epithelial Cells may Contribute to Aging-related Carcinogenesis. Cancer Research Communications, 2:772-783. 10.1158/2767-9764.CRC-22-0084 PMC10010334 36923312

[146] Dai L, Wang X, Bai T, Liu J, Chen B, Yang W (2022). Cellular Senescence-Related Genes: Predicting Prognosis in Gastric Cancer. Front Genet, 13:909546. 10.3389/fgene.2022.909546 PMC9198368 35719376

[147] Liu J, Zheng R, Zhang Y, Jia S, He Y, Liu J (2023). The Cross Talk between Cellular Senescence and Melanoma: From Molecular Pathogenesis to Target Therapies. Cancers (Basel), 15. 10.3390/cancers15092640 PMC10177054 37174106

[148] Dańczak-Pazdrowska A, Gornowicz-Porowska J, Polańska A, Krajka-Kuźniak V, Stawny M, Gostyńska A, et al. (2023). Cellular senescence in skin-related research: Targeted signaling pathways and naturally occurring therapeutic agents. Aging Cell, 22:e13845. 10.1111/acel.13845 PMC10265178 37042069

[149] Chojak R, Fares J, Petrosyan E, Lesniak MS (2023). Cellular senescence in glioma. J Neurooncol, 164:11-29. 10.1007/s11060-023-04387-3 37458855

[150] Salam R, Saliou A, Bielle F, Bertrand M, Antoniewski C, Carpentier C, et al. (2023). Cellular senescence in malignant cells promotes tumor progression in mouse and patient Glioblastoma. Nat. Commun, 14:441. 10.1038/s41467-023-36124-9 PMC9883514 36707509

[151] Cai X, Guillot A, Liu H. 2023. Cellular Senescence in Hepatocellular Carcinoma: The Passenger or the Driver? In Cells. 10.3390/cells12010132 PMC9818733 36611926

[152] Huang Y, Yang X, Meng Y, Shao C, Liao J, Li F, et al. (2021). The hepatic senescence-associated secretory phenotype promotes hepatocarcinogenesis through Bcl3-dependent activation of macrophages. Cell & Bioscience, 11:173. 10.1186/s13578-021-00683-5 PMC8447591 34530917

[153] Wang Y, Zhu H, Xu H, Qiu Y, Zhu Y, Wang X (2023). Senescence-related gene c-Myc affects bladder cancer cell senescence by interacting with HSP90B1 to regulate cisplatin sensitivity. Aging (Albany NY), 15:7408-7423. 10.18632/aging.204863 PMC10457043 37433010

[154] Santin Y, Lluel P, Rischmann P, Gamé X, Mialet-Perez J, Parini A (2020). Cellular Senescence in Renal and Urinary Tract Disorders. Cells, 9. 10.3390/cells9112420 PMC7694377 33167349

[155] Lewińska A, Przybylski P, Adamczyk-Grochala J, Błoniarz D, Litwinienko G, Wnuk M (2022). Senolysis-Based Elimination of Chemotherapy-Induced Senescent Breast Cancer Cells by Quercetin Derivative with Blocked Hydroxy Groups. Cancers (Basel), 14. 10.3390/cancers14030605 PMC8833762 35158873

[156] Özsoy Gökbilen S, Becer E, Vatansever HS (2022). Senescence-mediated anticancer effects of quercetin. Nutr. Res, 104:82-90. 10.1016/j.nutres.2022.04.007 35635900

[157] Kovacovicova K, Skolnaja M, Heinmaa M, Mistrik M, Pata P, Pata I, et al. (2018). Senolytic Cocktail Dasatinib+Quercetin (D+Q) Does Not Enhance the Efficacy of Senescence-Inducing Chemotherapy in Liver Cancer. Front Oncol, 8:459. 10.3389/fonc.2018.00459 PMC6218402 30425964

[158] Zhu Y, Tchkonia T, Fuhrmann-Stroissnigg H, Dai HM, Ling YY, Stout MB, et al. (2016). Identification of a novel senolytic agent, navitoclax, targeting the Bcl-2 family of anti-apoptotic factors. Aging Cell, 15:428-435. 10.1111/acel.12445 PMC4854923 26711051

[159] Skwarska A, Konopleva M (2023). BCL-xL Targeting to Induce Apoptosis and to Eliminate Chemotherapy-Induced Senescent Tumor Cells: From Navitoclax to Platelet-Sparing BCL-xL PROTACs. Cancer Res, 83:3501-3503. 10.1158/0008-5472.CAN-23-2804 37824434

[160] Mohamad Anuar NN, Nor Hisam NS, Liew SL, Ugusman A (2020). Clinical Review: Navitoclax as a Pro-Apoptotic and Anti-Fibrotic Agent. Front Pharmacol, 11:564108. 10.3389/fphar.2020.564108 PMC7768911 33381025

[161] Kumar RM, Kumar H, Bhatt T, Jain R, Panchal K, Chaurasiya A, Jain V. 2023. Fisetin in Cancer: Attributes, Developmental Aspects, and Nanotherapeutics. In Pharmaceuticals. 10.3390/ph16020196 PMC9961076 37259344

[162] Zhou C, Huang Y, Nie S, Zhou S, Gao X, Chen G (2023). Biological effects and mechanisms of fisetin in cancer: a promising anti-cancer agent. Eur. J. Med. Res, 28:297. 10.1186/s40001-023-01271-8 PMC10464434 37626424

[163] Yousefzadeh MJ, Zhu Y, McGowan SJ, Angelini L, Fuhrmann-Stroissnigg H, Xu M, et al. (2018). Fisetin is a senotherapeutic that extends health and lifespan. EBioMedicine, 36:18-28. 10.1016/j.ebiom.2018.09.015 PMC6197652 30279143

[164] Beltzig L, Christmann M, Dobreanu M, Kaina B (2024). Genotoxic and Cytotoxic Activity of Fisetin on Glioblastoma Cells. Anticancer Res, 44:901-910. 10.21873/anticanres.16884 38423634

[165] Muz B, Abdelghafer A, Markovic M, Yavner J, Melam A, Salama NN, Azab AK (2021). Targeting E-selectin to Tackle Cancer Using Uproleselan. Cancers (Basel), 13. 10.3390/cancers13020335 PMC7831123 33477563

[166] DeAngelo DJ, Jonas BA, Liesveld JL, Bixby DL, Advani AS, Marlton P, et al. (2022). Phase 1/2 study of uproleselan added to chemotherapy in patients with relapsed or refractory acute myeloid leukemia. Blood, 139:1135-1146. 10.1182/blood.2021010721 PMC11017789 34543383

[167] Ph K, Das S, G D, Mahanta N. 2022. Therapeutic Implications of Piperlongumine. In Handbook of Oxidative Stress in Cancer: Therapeutic Aspects. Chakraborti S., editor. Singapore: Springer Nature Singapore. 525-546.

[168] Liu X, Wang Y, Zhang X, Gao Z, Zhang S, Shi P, et al. (2018). Senolytic activity of piperlongumine analogues: Synthesis and biological evaluation. BMCL, 26:3925-3938. 10.1016/j.bmc.2018.06.013 PMC6087492 29925484

[169] Le HH, Cinaroglu SS, Manalo EC, Ors A, Gomes MM, Duan Sahbaz B, et al. (2021). Molecular modelling of the FOXO4-TP53 interaction to design senolytic peptides for the elimination of senescent cancer cells. EBioMedicine, 73:103646. 10.1016/j.ebiom.2021.103646 PMC8546421 34689087

[170] Allison SJ (2017). Targeting senescence-associated tissue damage. Nat. Rev. Nephrol, 13:319-319. 10.1038/nrneph.2017.53 28392563

[171] Baar MP, Brandt RMC, Putavet DA, Klein JDD, Derks KWJ, Bourgeois BRM, et al. (2017). Targeted Apoptosis of Senescent Cells Restores Tissue Homeostasis in Response to Chemotoxicity and Aging. Cell, 169:132-147.e116. 10.1016/j.cell.2017.02.031 PMC5556182 28340339

[172] Tripathi U, Chaib S, Gerdes EOW, Hogan KA, Zhu Y (2021). Development of a novel senolytic by precise disruption of FOXO4-p53 complex. EBioMedicine, 74:103693. 10.1016/j.ebiom.2021.103693 PMC8601985 34768086

[173] Zhang C, Xie Y, Chen H, Lv L, Yao J, Zhang M, et al. (2020). FOXO4-DRI alleviates age-related testosterone secretion insufficiency by targeting senescent Leydig cells in aged mice. Aging (Albany NY), 12:1272-1284. 10.18632/aging.102682 PMC7053614 31959736

[174] da Veiga Moreira J, Nleme N, Schwartz L, Leclerc-Desaulniers K, Carmona E, Mes-Masson A-M, Jolicoeur M. 2024. Methylene Blue Metabolic Therapy Restrains In Vivo Ovarian Tumor Growth. In Cancers. 10.3390/cancers16020355 PMC10814748 38254843

[175] Taldaev A, Terekhov R, Nikitin I, Melnik E, Kuzina V, Klochko M, et al. (2023). Methylene blue in anticancer photodynamic therapy: systematic review of preclinical studies. Front Pharmacol, 14:1264961. 10.3389/fphar.2023.1264961 PMC10568458 37841915

[176] dos Santos AF, Terra LF, Wailemann RAM, Oliveira TC, Gomes VdM, Mineiro MF, et al. (2017). Methylene blue photodynamic therapy induces selective and massive cell death in human breast cancer cells. BMC Cancer, 17:194. 10.1186/s12885-017-3179-7 PMC5353937 28298203

[177] Pawlowska E, Szczepanska J, Szatkowska M, Blasiak J (2018). An Interplay between Senescence, Apoptosis and Autophagy in Glioblastoma Multiforme-Role in Pathogenesis and Therapeutic Perspective. Int J Mol Sci, 19. 10.3390/ijms19030889 PMC5877750 29562589

[178] White E, Lowe SW (2009). Eating to exit: autophagy-enabled senescence revealed. Genes Dev, 23:784-787. 10.1101/gad.1795309 19339684

[179] Huang W, Hickson LJ, Eirin A, Kirkland JL, Lerman LO (2022). Cellular senescence: the good, the bad and the unknown. Nat Rev Nephrol, 18:611-627. 10.1038/s41581-022-00601-z PMC9362342 35922662

[180] Zhai J, Han J, Li C, Lv D, Ma F, Xu B (2023). Tumor senescence leads to poor survival and therapeutic resistance in human breast cancer. Front. oncol, 13:1097513. 10.3389/fonc.2023.1097513 PMC10019818 36937388

[181] Jaber S, Warnier M, Leers C, Vernier M, Goehrig D, Médard J-J, et al. (2023). Targeting chemoresistant senescent pancreatic cancer cells improves conventional treatment efficacy. Mol. Biol, 4:4. 10.1186/s43556-023-00116-4 PMC9899302 36739330

[182] Billimoria R, Bhatt P (2023). Senescence in cancer: Advances in detection and treatment modalities. Biochem. Pharmacol, 215:115739. 10.1016/j.bcp.2023.115739 37562510

[183] Yasuda T, Baba H, Ishimoto T (2023). Cellular senescence in the tumor microenvironment and context-specific cancer treatment strategies. FEBS J, 290:1290-1302. 10.1111/febs.16231 34653317

[184] Chen H-A, Ho Y-J, Mezzadra R, Adrover JM, Smolkin R, Zhu C, et al. (2023). Senescence rewires microenvironment sensing to facilitate antitumor immunity. Cancer Discov, 13:432-453. 10.1158/2159-8290.CD-22-0528 PMC9901536 36302222

[185] Wang C, Hao X, Zhang R (2022). Targeting cellular senescence to combat cancer and ageing. Mol Oncol, 16:3319-3332. 10.1002/1878-0261.13266 PMC9490146 35674055

[186] Fakhri S, Zachariah Moradi S, DeLiberto LK, Bishayee A (2022). Cellular senescence signaling in cancer: A novel therapeutic target to combat human malignancies. Biochem. Pharmacol, 199:114989. 10.1016/j.bcp.2022.114989 35288153

